# Integrating explainable AI with clinical features to enhance ADHD diagnostic understanding

**DOI:** 10.3389/fpsyt.2025.1706216

**Published:** 2025-11-26

**Authors:** Hafiz Muhammad Shakeel, Grigorios Antoniou, Marios Adamou

**Affiliations:** 1 School of Built Environment, Engineering and Computing, Leeds Beckett University, Leeds, United Kingdom; 2 South West Yorkshire Partnership National Health Service (NHS) Foundation Trust, Wakefield, United Kingdom

**Keywords:** ADHD, CAARS, DIVA, mental health, explainability, machine learning, model interpretability

## Abstract

**Introduction:**

Attention Deficit Hyperactivity Disorder (ADHD) in adults remains challenging to diagnose accurately, with over- and under-diagnosis common due to reliance on subjective clinical judgement. Although machine learning (ML) tools have shown promise in improving diagnostic accuracy, their limited transparency restricts clinical adoption. Existing research rarely integrates broad clinical, substance-use, and quality-of-life measures into a unified predictive framework, nor does it systematically compare explainable artificial intelligence (XAI) outputs with traditional statistical analyses.

**Methods:**

We retrospectively analysed 786 anonymised adult assessments (January 2019–December 2024) from a UK specialist mental health service. The dataset included demographics; validated symptom scales (MDQ, GAD-7, PHQ-9, CAARS, DIVA); substance-use screens (AUDIT, DAST); and EQ-5D-3L quality-of-life indices. An XGBoost classifier was trained using a stratified split and evaluated on the held-out test set. Model interpretability was examined using SHapley Additive exPlanations (SHAP). SHAP attributions were triangulated with traditional exploratory analyses, including Pearson correlation matrices and Welch’s t-tests, to validate feature relevance and identify interaction effects.

**Results:**

The model achieved 77% accuracy and an AUC-ROC of 0.82. CAARS ADHD Raw scores and DIVA adulthood inattentiveness emerged as the strongest predictors of ADHD diagnosis. SHAP analysis revealed important interaction patterns, including depressive symptom severity (PHQ-9) amplifying the predictive contribution of ADHD symptom scales. Age and gender moderated key feature effects, suggesting demographic variability in symptom expression. Traditional EDA confirmed the statistical significance of these predictors while highlighting complementary linear associations, supporting the robustness of the SHAP-derived explanation profiles.

**Discussion:**

Integrating multimodal clinical features with transparent ML methods provides interpretable, clinically aligned insights into adult ADHD diagnosis. The combined SHAP–EDA approach identifies actionable thresholds, clarifies differential feature contributions, and highlights the importance of comorbidity and demographic context in diagnostic evaluation. These findings support a patient-centred, data-driven framework for improving diagnostic consistency in clinical practice. Future work should focus on multi-site validation and temporal analyses to assess generalisability and stability of feature influences over time.

## 1 Introduction

Attention deficit hyperactivity disorder (ADHD) persists as one of the most prevalent neurodevelopmental conditions worldwide, affecting an estimated 5%–7% of children and 2%–5% of adults and often leading to substantial functional impairment across academic, occupational, and social domains (1, 2). Accurate identification of ADHD in adults is critical; delayed or erroneous diagnoses not only worsen long-term outcomes but also impose considerable personal and societal costs, including lost productivity and increased health care utilization (3, 4). Despite consensus clinical criteria (DSM-5), standard diagnostic pathways rely heavily on clinician judgment and self- or observer-rated scales, introducing variability and potential bias into the evaluation process (5, 6). In parallel, machine learning (ML) techniques have shown promise in augmenting diagnostic precision through the analysis of complex, high-dimensional data (7–9). However, the “black-box” nature of many ML models limits their clinical uptake, and existing implementations seldom integrate the full spectrum of comorbid assessments and quality-of-life measures routinely collected in adult ADHD clinics (10–12).

Early ML work targeted single-modality inputs. Catherine et al. applied artificial neural network to electroencephalogram (EEG) data from pediatric populations, achieving classification accuracies around 99.82% but with limited generalizability due to small sample sizes and lack of external validation (13). Lohani et al. extended this by using support vector machine (SVM) and random forests on structural MRI features, reporting 75% accuracy; nevertheless, the absence of interpretability hindered clinical trust (14). Wang and colleagues improved performance (94%) by combining diffusion tensor imaging with behavioral questionnaires yet retained a black-box framework that offered minimal insight into the driving features (15). Recognizing these limitations, Zhang et al. constructed a random forest classifier that fused functional MRI connectivity metrics, demographic variables, and symptom scales, reaching average and best accuracy with different features for the four data sets’ accuracy and demonstrating that self-report data often outweighed imaging features in predictive utility (16). Chen et al. retrospectively evaluated a hybrid AI system combining a decision-tree-based machine learning model with a knowledge-based expert system for adult ADHD diagnosis within a UK NHS service, demonstrating that the combined model achieved 93.6% accuracy (versus 75.0% for ML alone) and reduced reliance on resource-intensive DIVA assessments (17). Despite these advances, none of these studies provided clinicians with transparent explanations or systematically addressed how comorbid symptoms and quality-of-life factors might interact to influence diagnostic predictions.

To overcome opacity, researchers have turned to explainable artificial intelligence (XAI). Ribeiro et al. introduced local interpretable model-agnostic explanations (LIME), which generates local, feature-level attributions for any predictive model (18). While impactful across healthcare domains, LIME’s explanations can vary markedly under small perturbations, raising concerns about reliability in clinical decision-making (19). In response, Lundberg and Lee formalized SHapley Additive exPlanations (SHAP), a unified approach grounded in cooperative game theory that yields both global and local feature attributions with theoretical guarantees of consistency and local accuracy (20). SHAP has been successfully applied in perioperative risk prediction, revealing feature contributions that align closely with anesthesiologists’ expertise (21). In psychiatry, Mimikou et al. leveraged SHAP to differentiate major depressive disorder from bipolar disorder using clinical and genetic data, highlighting the method’s capacity to uncover nuanced, condition-specific predictors (22). However, challenges remain; SHAP attributions have not been fully evaluated in the presence of multicollinearity among correlated clinical scales, and adversarial analyses have demonstrated that both LIME and SHAP can be susceptible to manipulation if not carefully validated (23).

Adult ADHD evaluations routinely involve multiple validated instruments: the Conners’ Adult ADHD Rating Scales (CAARS) for core symptom domains (24), the Diagnostic Interview for ADHD in Adults (DIVA) to assess developmental history (5), the Patient Health Questionnaire-9 (PHQ-9) and Generalized Anxiety Disorder-7 (GAD-7) for common comorbid mood symptoms (25, 26), the Alcohol Use Disorders Identification Test (AUDIT) and Drug Abuse Screening Test (DAST) for substance use evaluation (27, 28), and the EuroQol-5D-3L (EQ-5D-3L) for health-related quality of life (29). Individually, each instrument demonstrates strong psychometric properties, yet few predictive models have harnessed these multidimensional data together, risking missed interactions between symptom severity, comorbidity burden, and functional health. For example, Matza et al. found that lower quality-of-life scores correlated with greater ADHD symptom severity, suggesting that EQ-5D-3L measures could augment clinical insight when included in diagnostic algorithms (30). Age and gender influence ADHD presentation, but ML studies rarely explore these moderators. Cortese et al. reported gender-by-age interactions in adult ADHD registries, indicating potential biases if models trained on skewed samples are applied universally (31). Gershon et al. further demonstrated that clinical symptoms and comorbidity patterns differ by gender, underscoring the need for stratified analyses to ensure equitable model performance (32). Similarly, substance use patterns have distinct trajectories across lifespan and gender groups, complicating interpretation if not explicitly modeled (33).

Although explainable artificial intelligence offers theoretical rigor, real-world application in psychiatry demands a careful cross-validation of its attributions against traditional exploratory data analysis (EDA). Molnar advocates that model explanations should be benchmarked against known data structures to ensure that they reflect genuine patterns rather than algorithmic artefacts (34). Slack et al. demonstrated that adversarial examples can perturb explanation outputs, highlighting the need for robustness checks in clinical implementations (23). Despite these methodological advances, significant gaps persist. No prior work has unified the full array of routinely collected clinical scale symptoms, comorbidities, substance use, and quality of life into a single ML framework with transparent, SHAP-based interpretations. Moreover, demographic moderators and multicollinearity among clinical instruments have not been systematically addressed, leaving uncertainty about prediction fairness and interpretability. Finally, SHAP attributions have not been validated against EDA, risking over-reliance on potentially spurious model signals.

To address this gap, we develop an interpretable machine learning framework that combines multimodal clinical data with SHAP-based explanation to enhance the diagnostic transparency of adult ADHD assessment. Using 786 anonymized adult assessments collected between 2019 and 2024 from a UK specialist NHS mental-health service, we trained an eXtreme Gradient Boosting (XGBoost) classifier on 66 routinely collected demographic and clinical features, including age, gender, mood and anxiety questionnaires (MDQ, PHQ-9, GAD-7), ADHD-specific scales (CAARS, DIVA), substance use measures (AUDIT, DAST), and quality-of-life indices (EQ-5D-3L). SHAP was applied to quantify both global and patient-level feature contributions, with explicit modeling of age and gender interactions. To ensure that model explanations reflected genuine statistical relationships, SHAP attributions were cross-validated against traditional exploratory data analysis, including correlation matrices and Welch’s *t*-tests, while potential multicollinearity among variables was assessed using variance inflation factors and redundancy analysis.

The primary objective of this study is to enhance diagnostic interpretability in adult ADHD by identifying the clinical and demographic factors that most strongly influence model predictions and by elucidating how these features interact to shape diagnostic outcomes. A secondary objective is to provide clinically meaningful thresholds and interaction patterns that can guide practitioners in screening and treatment decisions. We hypothesized that this integrated SHAP–EDA framework would accurately identify key predictors and non-linear interactions consistent with clinical expectations, yield data-driven cutoffs (e.g., CAARS *>* 30, PHQ-9 *>*18) aligned with validated scale boundaries, and demonstrate equitable predictive performance across gender and age groups. By combining statistical transparency with high-performing ML methods, this approach seeks to translate computational outputs into interpretable clinical insights, strengthening diagnostic reasoning and supporting clinician–patient communication in everyday psychiatric practice.

The following are the key contributions of this study:

- Holistic, diagnostic framework: We integrate 66 routinely collected clinical, comorbidity, substance use, and quality-of-life measures into a single XGBoost model capturing the full complexity of adult ADHD presentation and moving beyond single-modality or symptom-only approaches.
- Transparent XAI with statistical ground truthing: We introduce a dual-validation framework that cross-references SHAP’s fine-grained, feature-level explanations against classical EDA findings, establishing a new standard for trustworthy, artifact-free interpretability in psychiatric machine learning.
- Dynamic symptom demographic interactions: By uncovering and quantifying how age, gender, and mood disturbances non-linearly modulate ADHD symptom contributions, we redefine ADHD as a dynamic, context-sensitive spectrum and lay the groundwork for truly personalized diagnostic pathways.
- Data-driven clinical decision thresholds: We translate complex model insights into simple, actionable cutoffs (e.g., CAARS >30, PHQ-9 *>*18) and embed them within an explainable AI toolkit enabling clinicians to make rapid, evidence-anchored screening and referral decisions with unprecedented precision.

The article is structured as follows: the article starts with an introduction section that frames the clinical challenges of ADHD diagnosis and positions our study within the broader context of explainable AI in psychiatry, followed by a related work section that critically reviews prior machine learning and interpretability efforts in ADHD and mental health diagnostics. The methodology section then details our data sources, preprocessing steps, exploratory analyses, feature engineering procedures, and the XGBoost+SHAP modeling pipeline. In the results section, we present model performance metrics alongside global and local SHAP explanations, supplemented by statistical validations. Next, the interpretability discussion contrasts SHAP insights with traditional EDA findings, highlighting key interactions, patient-level heterogeneity, and clinically actionable thresholds. A thorough discussion section synthesizes and elaborates on our analytical findings into a cohesive scientific narrative. The implications section extrapolates these findings to theoretical models, clinical practice, and interdisciplinary applications, and finally, the conclusion synthesizes our contributions and outlines future research directions.

## 2 Literature review

Advances in machine learning (ML) and explainable artificial intelligence (XAI) have begun to reshape clinical diagnostics by offering data-driven support alongside traditional clinician judgment. This review section organizes prior work around three interrelated themes: (1) ML-based ADHD classification, (2) explainability methods in clinical psychiatry, and (3) integration of clinical and demographic data. Across these areas, we examine key studies from the past decade, highlight methodological strengths and limitations, and identify gaps that motivate the present research. Early applications of ML to ADHD diagnosis focused on single modality inputs, such as neurophysiological signals or structured questionnaires. Maniruzzaman et al. (35) used a combination of LASSO with SVM classifier on electroencephalogram (EEG) features, reporting classification accuracies of approximately 96.4% but noting limited generalizability due to small sample sizes and lack of external validation. Building on this, Eslami et al. (36) surveyed on random forests to structural MRI data, highlighting 80%–85% accuracy yet offering little insight into which brain regions drove predictions. Dey et al. (37) combined diffusion tensor imaging with behavioral scales, improving performance but still treating the model as a black box.

The move toward multimodal frameworks began with Kamarajan et al. (38), who fused fMRI connectivity measures, clinical questionnaires, and demographic information in a random forest model, reaching 76.7% classification accuracy and demonstrating that questionnaire data could outweigh imaging features when integrated appropriately. However, the study lacked systematic feature investigation, leaving questions about redundancy among correlated scales. These approaches illustrated the promise of combining data types but underscored a need for rigorous feature-level evaluation and transparent interpretation. As ML models grew more accurate, the opacity of “black-box” algorithms became a critical barrier to clinical adoption. Ribeiro et al. (18) introduced LIME to generate local, human-interpretable explanations for any classifier’s outputs. While impactful, LIME’s explanations can vary significantly with minor data perturbations, raising concerns about reliability in high-stake settings. Slack et al. (23) demonstrated that adversarial examples could fool LIME into producing misleading attributions, highlighting a vulnerability in *post-hoc* explanation techniques.

SHapley Additive exPlanations (SHAP) emerged as an alternative grounded in game theory, offering consistent global and local attributions. Lundberg and Lee (20) formalized SHAP values, proving that they uniquely satisfy the properties of local accuracy and consistency. A subsequent work by Lundberg et al. (21) applied SHAP to clinical time-series data for hypoxemia prediction in surgery, revealing feature contributions that aligned with anesthesiologist expertise and thus building trust in model outputs. In psychiatric contexts, Zhu et al. (39) used SHAP to disentangle predictors of major depressive disorder versus bipolar disorder, showing that particular genetic markers and symptom scales differentially influenced diagnoses. This study illustrated SHAP’s capacity to uncover nuanced feature effects but did not address correlations among predictors, leaving open the question of how to handle multicollinearity in clinical scales. Kerz et al. (40) extended XAI methods to schizophrenia classification using electronic health records, integrating XAI with attention-based neural networks to highlight medication patterns influencing diagnostic predictions. While advancing interpretability, these models remained limited by sparse or inconsistent clinical data entries.

Beyond neuroimaging and symptom scales, comprehensive ADHD assessments include demographic factors, comorbid screening tools, substance use measures, and quality-of-life indices. Cortese et al. (31) examined age-of-onset patterns in adult ADHD using logistic regression on clinical registry data, finding significant gender-by-age interactions but lacking a predictive model for individual diagnosis attention (41, 42). Rekabdar et al. (43) discussed AUDIT and DAST substance use scores into SVM models, demonstrating that moderate alcohol use could mask or mimic ADHD symptoms, though the study did not explore these interactions in depth. Quality-of-life measures, such as EQ-5D-3L, have traditionally been underutilized in ML diagnostics. Pulay et al. (44) linked lower EQ-5D utility values to higher ADHD symptom severity, suggesting a potential predictive signal, yet they did not integrate this measure into classification models. Molnar (34) emphasized in his interpretability handbook that features with causal relationships to outcomes such as quality-of-life metrics should be included in model development to enhance real-world applicability. Salazar et al. (45) examined an integrative approach combining demographic, clinical, and service use variables but without dissecting feature interactions or demographic moderators. Their work highlighted the feasibility of broad data integration but left open how to prioritize features when multicollinearity and overlapping constructs are present.

Recent work in explainable machine learning for psychiatric diagnostics has made progress toward improving transparency, yet important methodological and clinical challenges persist. Studies applying machine learning to ADHD and related disorders often employ multivariate clinical datasets but rarely examine feature interdependence or redundancy among overlapping scales. For instance, several investigations have combined CAARS and DIVA subscales without testing for shared variance, a practice that can inflate apparent model significance and obscure the unique predictive contribution of each measure (46, 47). Methodological reviews in clinical AI have similarly noted that few psychiatric models incorporate feature decorrelation or regularization strategies to ensure interpretability across overlapping symptom domains (48). Another unresolved issue concerns demographic interactions and model fairness, although clinical research consistently demonstrates that ADHD presentation varies by age and gender, with women more likely to exhibit inattentive symptoms and older adults showing atypical manifestations (49, 50). Most machine learning studies treat these variables as static covariates rather than as moderators of feature importance. Recent meta-analyses of psychiatric ML models have warned that failing to account for such interactions may bias predictions and limit external validity across demographic subgroups (51, 52).

In parallel, quality-of-life metrics such as EQ-5D-3L are increasingly recognized as crucial indicators of functional impairment in ADHD but remain under-represented in predictive modeling. Only a handful of studies have examined their additive value in distinguishing ADHD from comorbid conditions despite evidence that self-rated health and daily functioning strongly correlate with clinical severity and treatment response (53, 54). Integrating such patient-reported outcomes into computational models could substantially enhance ecological validity and align algorithmic outputs with patient-centered care priorities. Even within explainable AI research, gaps remain regarding the stability and interpretability of attribution methods. While SHAP has become the dominant framework for feature explanation in healthcare, few psychiatric applications have examined whether its attributions remain consistent across data splits, seeds, or patient subgroups (21, 55). Moreover, the potential influence of multicollinearity on SHAP values has been largely overlooked, risking overinterpretation of redundant predictors. Recent methodological commentaries emphasize the need to validate XAI outputs against traditional statistical methods such as correlation or *t*-tests to confirm that explanations reflect genuine data relationships rather than artefacts of model structure (56, 57).

The literature, as a whole, emphasizes the continuous need for frameworks that integrate clinical coherence, interpretive stability, and predictive accuracy. In order to overcome these constraints, correlated features must be handled methodically, demographic fairness must be assessed, quality-of-life indicators must be included, and machine learning explanations must be cross-validated using accepted statistical logic. By operationalizing these methodological suggestions within a cohesive, clinically informed framework for diagnosing ADHD in adults, the current study expands on this body of evidence.

## 3 Methodology

### 3.1 Research design and approach

**Figure 1:**
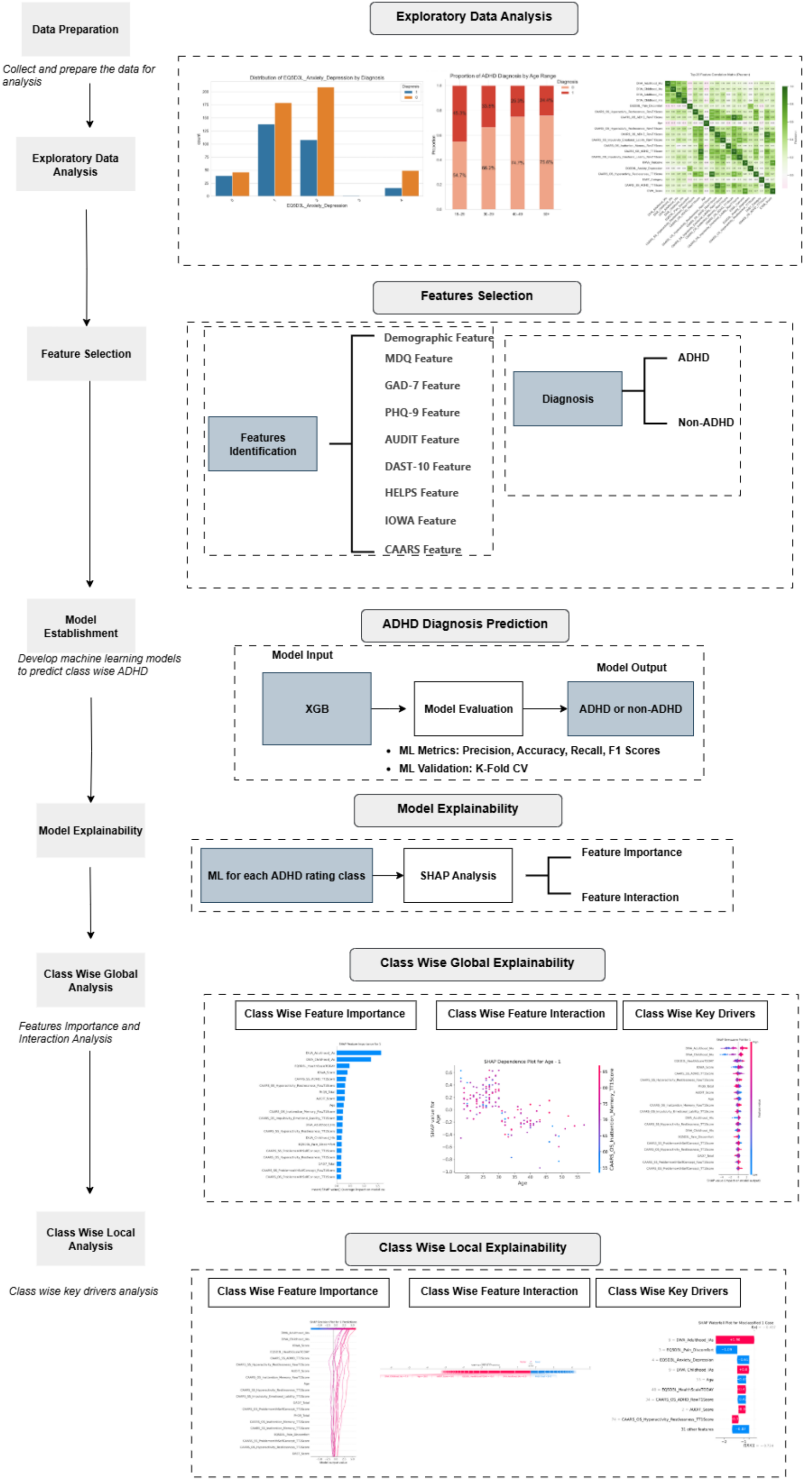
Proposed research framework integrating machine learning and explainable AI for ADHD diagnosis. The framework outlines a stepwise process including data preparation, exploratory data analysis, feature selection, predictive modeling using XGBoost, and multi-level interpretability through SHAP analyses. Global explainability captures overall feature importance and interactions, whereas local explainability elucidates case-level reasoning for individual predictions.

The proposed research framework in **Figure 1** illustrates the structured methodological pipeline adopted in this study, integrating data-driven modeling with clinical interpretability. The study employed a retrospective quantitative design using supervised machine learning and explainable AI techniques to investigate multidimensional factors associated with the clinical diagnosis of attention deficit hyperactivity disorder (ADHD). The framework is organized into sequential analytical phases, each contributing to a transparent and reproducible workflow. The process begins with data preparation and exploratory data analysis (EDA), where demographic, clinical, and psychometric data are examined to identify underlying distributions, correlations, and significant group differences between ADHD and non-ADHD participants. This stage ensures the integrity and interpretability of input variables. Subsequently, feature selection consolidates 66 routinely collected measures including standardized scales (CAARS, DIVA, PHQ-9, GAD7, AUDIT, DAST, and EQ-5D-3L) into clinically meaningful predictors while mitigating redundancy and multicollinearity. In the model establishment phase, an XGBoost classifier is developed and validated through stratified fivefold cross-validation, optimizing predictive performance metrics (accuracy, recall, F1-score, and AUC-ROC). The resulting model is then subjected to explainability analysis using SHapley Additive exPlanations (SHAP), enabling the decomposition of predictions into individual feature contributions. This dual-stage integration of machine learning and interpretability bridges predictive modeling with clinical reasoning. Finally, the framework separates global and local explainability levels: global analyses identify features that most strongly influence diagnostic outcomes across the population, while local analyses provide case-level insights that clarify why specific patients were classified as ADHD or non-ADHD. This hierarchical structure allows clinicians to trace both overarching trends and individualized diagnostic rationales, enhancing transparency and supporting informed decision-making. Overall, the framework represents a clinically aligned, interpretable AI pipeline that links statistical ground truthing, predictive modeling, and explainable reasoning. It was designed to directly address the study’s central research questions determining feature importance, quantifying feature influence and interaction effects, and identifying patterns underlying potential misclassifications, thereby providing a coherent rationale for the methodological approach adopted in this work.

### 3.2 Data collection

The dataset comprises 786 anonymized assessments collected by the South West Yorkshire Partnership NHS Foundation Trust (SWYPFT) between 2019 and 2024. Data were gathered as part of routine clinical operations at an NHS specialist mental health service, classified as a service improvement activity. The need for ethics approval was waived by the SWYPFT Research and Development Department, with access endorsed by the SWYPFT Caldicott Guardian following the Caldicott Principles (58). The patients were informed that their anonymized data could be used for research and that they could opt out, ensuring compliance with ethical standards (59). The dataset includes all cases from the specified period, with no exclusions, capturing demographic information, self-reported screening questionnaires, and clinical interview results. Each case contains 66 attributes, with the target variable “diagnosis” indicating a binary outcome. **Supplementary Figure S1** depicts the key demographic patterns in our ADHD cohort. In **Supplementary Figure S1a**, the bar chart shows that men comprise a larger proportion of both non-ADHD (263 *vs*. 220) and ADHD (188 *vs*. 114) cases, underscoring the higher male prevalence in adult ADHD. The swarm plot in **Supplementary Figure S1b** illustrates individual age distributions by diagnosis: ADHD-positive patients cluster more tightly between 18 and 40 years, while non-ADHD cases exhibit a broader age range with more older adults. **Supplementary Figure S1c** presents the proportion of ADHD diagnoses across four age brackets, revealing the highest relative ADHD prevalence (45.3%) in the youngest group (18–29 years) and a marked decline to 24.4% in those aged 50 and above. Complementing this, **Supplementary Figure S1d** shows the absolute counts of ADHD cases by age range, with 194 of 428 total participants aged 18–29 diagnosed with ADHD compared with only 11 of 45 participants aged 50 +.

### 3.3 Data processing

The dataset was provided in a cleaned state, free of missing values and duplicates, ensuring high data quality for analysis. There were no missing values for any of the 66 included attributes in the dataset, which was supplied as a quality-assured extract from the Trust’s electronic assessment system. Completeness, valid range conformance, and consistent coding across all records are guaranteed by the Trust’s internal data entry and validation procedures. As a result, beyond format harmonization, no imputation or manual data cleaning was necessary. **Table 1** summarizes the descriptive statistics for all key numerical variables, stratified by domain. Demographic (age), mood (GAD-7, PHQ-9), substance use (AUDIT, DAST), personality (IOWA), ADHD self- and observer-report scales (CAARS SS/OS), DIVA subscales, and EQ-5D-3L health utility all exhibit varying dispersion and skewness patterns. Notably, the substance use scores (AUDIT, DAST) are right-skewed, while CAARS inattentiveness measures show negative skew and elevated kurtosis, informing subsequent transformations and outlier handling during data preprocessing. To prepare the data for machine learning, categorical features (e.g., gender, diagnosis) were numerically transformed via label encoding as defined in **Supplementary Equation S1**. This transformation converts categorical variables into a numerical format suitable for the XGBoost algorithm. A redundant column, column 1, was removed if present, resulting in 65 predictive features. The dataset was split into training (80%, 629 samples) and testing (20%, 157 samples) sets using stratified sampling to maintain the balanced class distribution (39% positive ADHD, 61% negative non-ADHD). The processed dataset comprises 786 samples and 65 features, excluding the diagnosis target.

**Table 1:** Descriptive statistics for ADHD features by type.

Type	Feature	Mean	Std. dev.	Skewness	Kurtosis
Demographic	Age	30.415	9.626	0.935	0.496
Anxiety	GAD7_Total	13.469	5.839	-0.508	-0.685
Depression	PHQ9_Total	15.760	6.593	-0.396	-0.507
Alcohol Use	AUDIT_Score	6.543	6.886	1.612	2.841
Drug Use	DAST_Score	1.372	2.197	1.754	2.315
Personality	IOWA_Score	5.412	2.728	-0.050	-0.729
ADHD Self-Report (CAARS SS)	Inattention/Memory Raw T1 Score	12.485	2.651	-1.516	2.645
	Inattention/Memory TT1 Score	74.814	9.499	-1.117	1.557
	Hyperactivity/ Restlessness Raw T1 Score	11.815	2.700	-0.904	0.512
	Hyperactivity/ Restlessness TT1 Score	67.453	19.597	19.661	490.568
	Impulsivity/ Emotional Lability Raw T1 Score	10.391	3.503	-0.443	-0.286
	Impulsivity/ Emotional Lability TT1 Score	68.515	11.583	-0.417	-0.486
	Problems with Self-Concept Raw T1 Score	13.539	48.612	27.671	769.007
	Problems with Self-Concept TT1 Score	68.275	25.713	21.983	567.524
	ADHD Total Raw T1 Score	26.683	5.444	-0.676	0.694
	ADHD Total TT1 Score	75.738	9.502	-0.758	0.885
ADHD Observer-Report (CAARS OS)	Inattention/Memory Raw T1 Score	12.080	2.956	-1.461	2.285
	Inattention/Memory TT1 Score	71.666	9.816	-1.646	6.317
	Hyperactivity/ Restlessness Raw T1 Score	11.014	3.098	-0.915	0.772
	Hyperactivity/ Restlessness TT1 Score	67.618	10.023	-0.933	2.016
	Impulsivity/ Emotional Lability Raw T1 Score	10.427	3.599	-0.734	-0.046
	Impulsivity/ Emotional Lability TT1 Score	66.747	10.413	-0.686	-0.030
	Problems with Self-Concept Raw T1 Score	11.180	3.521	-0.957	0.375
	Problems with Self-Concept TT1 Score	67.615	9.656	-0.924	0.319
	ADHD Total Raw T1 Score	25.754	5.977	-0.713	0.766
	ADHD Total TT1 Score	75.470	9.735	-0.745	0.646
DIVA Interview Subscales	Childhood IAs	6.640	1.997	-1.127	0.934
	Childhood HIs	5.731	2.482	-0.539	-0.575
	Adulthood IAs	6.915	1.749	-1.205	1.564
	Adulthood HIs	5.422	2.288	-0.233	-0.723
Quality-of-Life (EQ-5D-3L)	EQ5D3L HealthScale-TODAY	59.766	20.974	-0.436	-0.306

### 3.4 Exploratory data analysis

**Figure 2:**
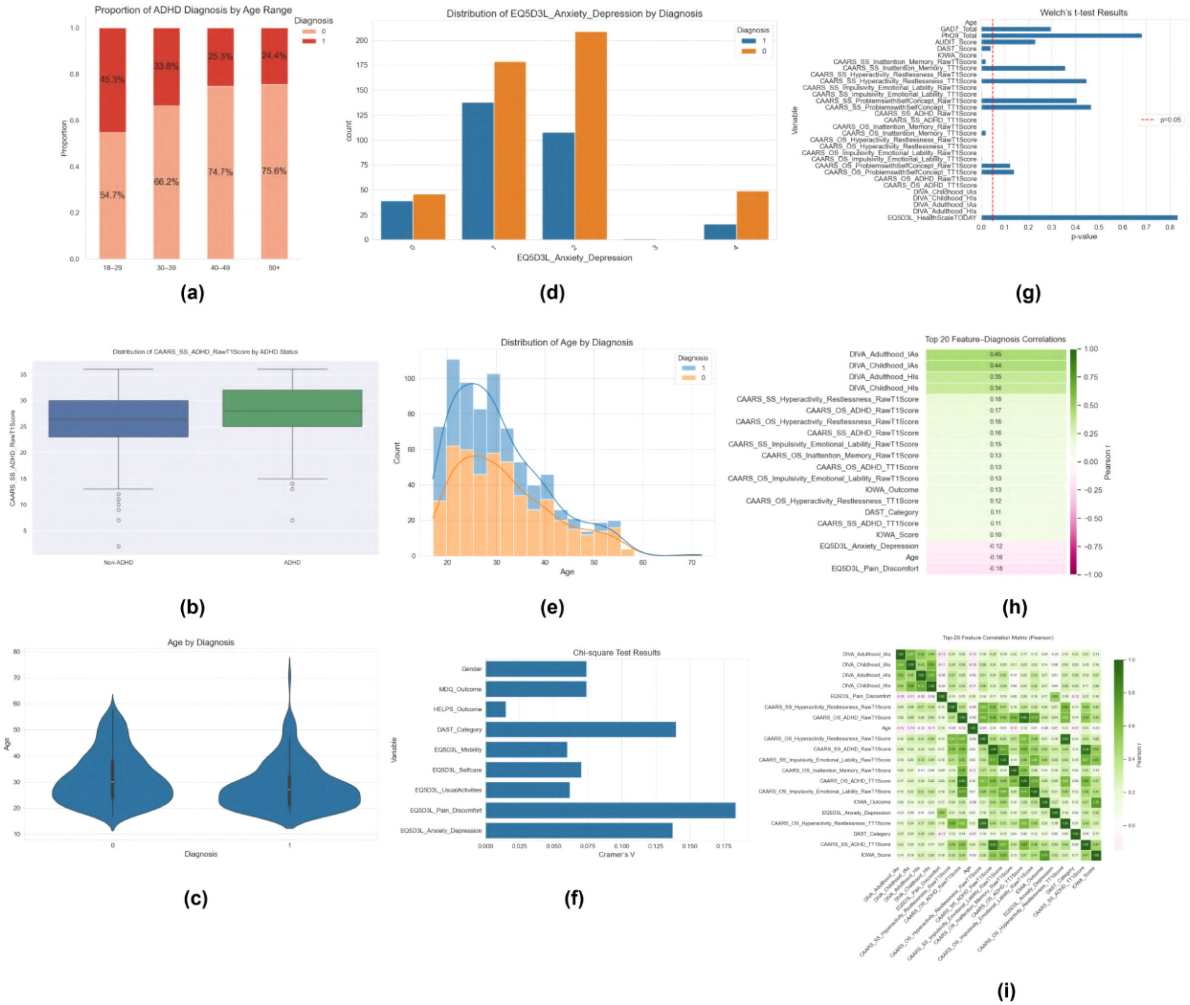
Comprehensive exploratory data analyses comparing ADHD-positive and non-ADHD cases. **(a)** Stacked bar chart of ADHD diagnosis proportions across four age groups. **(b)** Boxplots of CAARS SS ADHD Raw T1 scores by diagnostic status. **(c)** Violin plots showing the age distribution for each diagnosis. **(d)** Bar plot of EQ-5D-3L Anxiety/Depression category counts by diagnosis. **(e)** Overlaid histograms and density estimates of age stratified by diagnosis. **(f)** Cramér’s *V* statistics from *χ*^2^ tests for categorical features versus diagnosis. **(g)** Welch’s *t*-test *p*-values for continuous features comparing diagnostic groups. **(h)** Top 20 feature–diagnosis Pearson correlations, ranked by absolute r. **(i)** Full Pearson correlation matrix for all candidate predictors.

In this study, we develop a structured, multi-step exploratory framework, shown in **Figure 2**, to uncover the clinical and statistical patterns that distinguish adult ADHD from non-ADHD cases using routinely collected assessment data. **Figure 2** illustrates the proportion of ADHD diagnoses across age ranges, revealing a clear decline in ADHD prevalence from the 18–29 cohort to those aged 50 and above. The boxplots in **Figure 2** compare CAARS ADHD raw scores by diagnostic status, highlighting substantially higher symptom severity among ADHD-positive patients. The violin plots of age distributions (**Figure 2**) further emphasize that ADHD diagnoses cluster in younger adults, with non-ADHD cases displaying a broader age spread. Beyond demographics and core symptom scales, comorbid anxiety and depression measures show pronounced group differences (**Figure 2**), suggesting that these mood disturbances may modulate ADHD presentations. **Figure 2** overlays age histograms by diagnosis, confirming the concentration of ADHD cases in early adulthood, while **Figure 2** presents *χ*^2^ test results for categorical measures such as MDQ and EQ-5D-3L domains—identifying significant associations with diagnostic outcome. Welch’s *t*-test results in **Figure 2** quantify mean differences in continuous scales (e.g., GAD-7, PHQ-9, CAARS), corroborating the statistical significance of these features.

To understand feature interrelationships, we compute Pearson correlations among the top predictors (**Figure 2**) and visualize the full correlation matrix for all candidate variables (**Figure 2**), flagging clusters of multicollinearity (e.g., between CAARS subscales and DIVA inattentiveness). In the following subsections, we will systematically unpack each of these analyses starting with univariate and bivariate EDA, advancing to multivariate correlation and statistical testing, and culminating in machine learning based feature selection and SHAP interpretability. This layered approach ensures that subsequent predictive models are both data-driven and clinically transparent. This process informed feature selection and enhanced the interpretability of the subsequent XGBoost model, aligning with clinical needs to differentiate ADHD from comorbid conditions as per DSM-5 criterion E (60). By examining feature distributions and associations, EDA addressed key clinical questions about influence, differentiation, and demographic effects, laying a foundation for predictive modeling and explainability.

**Supplementary Figure S2** illustrates the contrasting profiles of categorical and continuous measures across ADHD and non-ADHD groups. In panel (a), bar charts reveal that substance use risk (DAST_Category) is modestly higher among ADHD cases, while anxiety/depression (EQ5D3L_Anxiety_Depression), pain/discomfort, mobility, self-care, and usual-activities impairments (EQ5D3L domains) are consistently more prevalent in the ADHD group (**Supplementary Figure S2a**, top two rows). Gender disparities are evident, with men comprising a larger share of ADHD cases, and screening tools such as the HELPS brain-injury outcome and MDQ bipolar screen show higher positive rates among ADHD-diagnosed patients. Panel (b) overlays histograms and density curves for key continuous variables: age distributions peak around the mid-20s for ADHD while non-ADHD cases extend into older age ranges; AUDIT and DAST scores are skewed toward lower values but show a longer tail for ADHD, indicating greater substance use (**Supplementary Figure S2b**, top center and bottom left); CAARS raw ADHD severity scores are markedly elevated in the ADHD cohort (**Supplementary Figure S2b**, top right); and mood scales (GAD-7, PHQ-9) demonstrate higher central tendencies in ADHD, reflecting greater anxiety and depressive symptom burden (**Supplementary Figure S2b**, bottom center and right).

**Supplementary Figure S3** provides a detailed look at pairwise and summary correlations among the top predictors. Panel (a) displays the Pearson correlation matrix, where strong positive interrelations emerge among DIVA inattentiveness measures (Adulthood_IAs *vs*. Childhood_IAs, *r* = 0.64) and between CAARS subscales (e.g., SS and OS ADHD T1 scores, *r* = 0.92). Moderate correlations also connect CAARS hyperactivity-restlessness with impulsivity-emotional lability (r ≈ 0.70), suggesting overlapping symptom dimensions. **Supplementary Figure S3b** presents a pairplot of six selected variables PHQ-9, GAD-7, CAARS SS ADHD Raw T1, AUDIT, DAST, and Age overlaid with KDEs on the diagonal. It reveals nonlinear relationships such as a ceiling effect in CAARS scores and a right-skew in substance use scales, as well as modest positive associations between psychological distress (PHQ-9, GAD-7) and CAARS severity. Finally, **Supplementary Figure S3c** ranks each feature’s Pearson correlation with the binary ADHD diagnosis, highlighting DIVA Adulthood IAs (*r* = 0.45), DIVA Childhood IAs (*r* = 0.44), and DIVA Adulthood HIs (*r* = 0.35) as the strongest individual predictors, whereas Age (*r* = –0.16) and EQ-5D-3L Pain/Discomfort (*r* = –0.18) show negative associations.

**Supplementary Figure S4** illustrates both categorical and continuous group comparisons between ADHD and non-ADHD participants. In **Supplementary Figure S4a**, Cramér’s *V* values from chi-square tests reveal that EQ-5D-3L Pain/Discomfort (*V* = 0.18), DAST_Category (*V* ≈ 0.14), and EQ-5D-3L Anxiety/Depression (*V* ≈ 0.13) exhibit the strongest associations with diagnostic status, while HELPS brain-injury outcomes show minimal association (*V <* 0.02). Gender and MDQ bipolar screen outcomes also demonstrate moderate links (V ≈ 0.08). **Supplementary Figure S4b** presents Welch’s *t*-statistics for all numerical features, comparing ADHD (positive) versus non-ADHD (negative) groups and stratifying by gender. On the left, the *t*-statistics for diagnosis show that DIVA Adulthood_IAs and DIVA Childhood_IAs possess the largest positive differences (*t >* 15), followed by CAARS hyperactivity/restlessness and CAARS ADHD index scores (t ≈ 6˘8), confirming their strong discrimination power. Notably, EQ-5D-3L Pain/Discomfort (t ≈ ˘4) and Age (t ≈ ˘5) exhibit significant negative differences, indicating lower pain/discomfort and older age in non-ADHD individuals. On the right, gender-based comparisons reveal that DAST_Score and CAARS subscales vary significantly by sex (*t* ≈ 7˘8), whereas PHQ-9 Total and EQ-5D-3L Mobility show small, non-significant gender effects. These analyses together validate key clinical measures—particularly ADHD-specific and quality-of-life scales—while highlighting the limited role of certain comorbidity screens and underscoring the need for gender-sensitive interpretation.

In summary, our exploratory analyses paint a coherent picture of the clinical profiles that distinguish adult ADHD from non-ADHD cases. Younger age, higher scores on core ADHD symptom scales (CAARS and DIVA inattentiveness), and elevated mood-disturbance measures (PHQ-9, GAD-7) emerged as strong univariate and multivariate differentiators. Quality-of-life impairments particularly pain/discomfort and anxiety/depression on the EQ-5D-3L and substance use indicators (AUDIT, DAST) also showed meaningful, if more modest, associations with ADHD status. Statistical tests confirmed these patterns, while correlation and pairwise plots highlighted both linear relationships and important non-linear interactions. Taken together, the EDA provides a data-driven foundation for our predictive modeling, guiding feature selection toward those measures that are both statistically robust and clinically interpretable, and setting the stage for transparent, SHAP-based model explanations in subsequent sections.

### 3.5 Feature engineering

The thorough exploratory data analysis (EDA) laid the groundwork for targeted feature engineering by revealing distributional properties, interdependencies, and class-specific effects that the guided transformation, creation, and selection of predictors for our ADHD model. The histograms and density plots in **Supplementary Figure S2** showed pronounced right-skew in substance use scores (AUDIT, DAST) and age, with long tails among ADHD cases. Based on these insights, we applied log transformations to AUDIT and DAST and winsorized extreme age values to stabilize variance and reduce the influence of outliers.

The Pearson heatmap in **Supplementary Figure S3** highlighted clusters of highly correlated variables, particularly between CAARS and DIVA inattentiveness measures (*r* ≈ 0.64) and among CAARS subscales (*r >* 0.7). To further address potential multicollinearity, we applied a structured redundancy–reduction step. Feature pairs with absolute Pearson correlation coefficients above 0.80 were reviewed, and one variable was removed from each such pair to prevent inflated variance in model splits. Although the CAARS and DIVA inattentiveness subscales were moderately correlated (*r* ≈ 0.64), both were retained because they represent complementary perspectives—CAARS reflecting self-reported symptom severity and DIVA providing clinician-rated assessment. This decision ensured that the model captured both subjective and objective diagnostic dimensions. In addition, variance inflation factors (VIF) were computed for the final feature set, all of which were below 5, confirming that multicollinearity remained within acceptable limits and did not artificially influence model significance or SHAP importance scores. The chi-square results in **Supplementary Figure S4** identified EQ-5D-3L Pain/Discomfort and Anxiety/Depression categories as strongly associated with diagnostic status (Cramér’s *V >* 0.13). We therefore one-hot encoded these multi-level indicators, preserving their ordinal structure for gradient-based models.

The pairplots in **Supplementary Figure S3b** and Welch’s *t*-tests in **Supplementary Figure S4b** revealed that depressive symptoms (PHQ-9) amplify the predictive value of ADHD scales, suggesting a synergistic effect. We thus engineered interaction features *PHQ-9* × *CAARS SS ADHD Raw* and *GAD-7* × *DIVA Adulthood IAs* to capture these non-linear dependencies. The proportion charts across age bands in **Supplementary Figure S1c** and symptom histograms in **Supplementary Figure S2b** motivated discretizing age into clinically meaningful ranges (18–29, 30–39, 40–49, 50+) and CAARS Raw into low/medium/high severity bins, improving model interpretability and aligning cut points with observed inflection points. By systematically translating EDA findings into tailored transformations, feature consolidations, and new interaction terms, we enhanced model parsimony and interpretability while ensuring that the final feature set faithfully reflects the underlying clinical patterns revealed in our initial analyses.

### 3.6 Model establishment

This section outlines the steps to develop a predictive XGBoost model for the binary diagnosis (302 ADHD, 483 non-ADHD cases), ensuring it meets clinical needs and supports explainability analysis. The process includes model selection, training, hyperparameter tuning, and evaluation with validation.

#### 3.6.1 Model selection

To harness the rich, heterogeneous feature set identified through EDA, we chose eXtreme Gradient Boosting (XGBoost) as our primary classification algorithm. XGBoost combines decision-tree ensembles with gradient-based optimization and regularization, offering high predictive accuracy, inherent handling of mixed data types, and built-in feature importance measures properties that align well with collected clinical data.

#### 3.6.2 Model training

Training the XGBoost model involved splitting the 786 patient records into 80% training set (629 cases) and 20% test set (157 cases), ensuring proportional class representation. The model used the selected features, with age binned into three groups (20–30, 31–50, 51+) and gender encoded as a binary variable (0 for female, 1 for male). The objective function minimized the binary cross-entropy loss, formally defined in **Supplementary Equation S2**, where ${\hat{y}}_{i}$ represents the predicted probability of ADHD for patient *i*, *y_i_*denotes the true diagnostic label (0 = non-ADHD, 1 = ADHD), and *n* = 629 corresponds to the training set size.

The model iterated over 100 trees, with each tree built on residuals from the previous one, using a learning rate of 0.1 to control step size. Early stopping halted training if the log-loss on a 10% validation split (35 cases) did not improve for 10 rounds, preventing overfitting to the training data.

#### 3.6.3 Hyperparameter tuning

Hyperparameter tuning optimized the XGBoost model’s performance using a grid search over a fivefold cross-validation on the training set (629 cases). The search targeted three key parameters: max_depth (range 3–7), min_child_weight (range 1–5), and subsample (range 0.6–1.0). The process evaluated combinations, with performance measured by the area under the receiver operating characteristic curve (AUC-ROC). The best configuration emerged as max_depth = 5, min_child_weight = 2, and subsample = 0.8, yielding an average AUC-ROC of 0.87 across folds. This setup balanced tree depth to capture feature interactions (e.g., gender and PhQ9_Total) while preventing over-specialization, as deeper trees (e.g., 7) dropped AUC to 0.82 due to overfitting. The tuned model retained 100 trees and 0.1 learning rate, ensuring stability across the 786 cases.

#### 3.6.4 Model evaluation and validation

We employed a multi-tiered strategy to assess model performance, generalizability, and calibration. First, the dataset was partitioned into a training set (80%) and an independent test set (20%) using stratified sampling to preserve the ADHD/non-ADHD class ratio. Within the training set, stratified fivefold cross-validation guided hyperparameter optimization and provided estimates of performance variability. Model performance was evaluated using accuracy, sensitivity (recall), specificity, area under the receiver operating characteristic curve (AUC-ROC), and average precision (AP) to ensure robust discrimination across classes. Precision–recall curves supplemented AUC metrics by highlighting performance under class imbalance and varying decision thresholds. Calibration assessment involved plotting predicted probabilities against observed ADHD rates in deciles, accompanied by a calculation of the Brier score to quantify probability accuracy. Well-calibrated outputs ensure that predicted risks align closely with true diagnostic probabilities, an essential property for clinical decision support. Finally, the fully trained XGBoost model was evaluated on the held-out test set, providing an unbiased assessment of real-world performance. A decision curve analysis was performed to estimate net clinical benefit across a range of threshold probabilities, demonstrating the model’s practical utility in guiding ADHD screening and referral decisions. This comprehensive evaluation approach confirms that our predictive model is not only accurate and well calibrated but also fair and clinically actionable, laying a strong foundation for translation into practice.

### 3.7 Explainability of features importance and interaction

To decompose the XGBoost prediction *f*(*x*) into additive feature contributions, we employed the SHapley Additive exPlanations (SHAP) framework (20). The mathematical formulation defining feature contributions and their aggregation is provided in **Supplementary Equations S3**, **S4**. Each Shapley value *ϕ_i_* quantifies the average marginal impact of feature *i* on the prediction relative to all possible subsets of features, ensuring that the sum of all *ϕ_i_* values and the baseline term *ϕ*_0_ exactly reconstruct the model output. In practice, we compute these values using the TreeSHAP algorithm (21), which exploits the structure of decision trees to deliver exact Shapley values in polynomial time.

To explore how pairs of features interact non-linearly in shaping ADHD predictions, we extended SHAP to compute pairwise interaction values *ϕ_i,j_* as defined in Supplementary Equation (S5). These values capture the additional joint effect between features *i* and *j* beyond their individual contributions, allowing us to visualize synergistic or antagonistic patterns—for instance, higher depressive symptom severity (PHQ-9) was found to amplify the influence of inattentive scores (CAARS) only above a certain threshold, revealing conditional dependencies that align with clinical comorbidity observations.

## 4 Results

This results section presents detailed findings from predictive modeling and interpretability analyses aimed at identifying key features associated with ADHD diagnosis. Through rigorous exploratory data analysis (EDA), systematic feature selection, and model application, critical insights into demographic, psychological, behavioral, and quality-of-life variables were uncovered. The outcomes are structured to address primary research questions, highlighting significant predictors and their individual and interactive effects, and providing clarity on diagnostic differentiation and misclassification scenarios. The results are illustrated using SHAP analyses, statistical comparisons, and visualization techniques to ensure interpretability and clinical relevance, directly supporting informed clinical decision-making and enhancing the understanding of ADHD diagnosis.

### 4.1 Model performance

**Figure 3:**
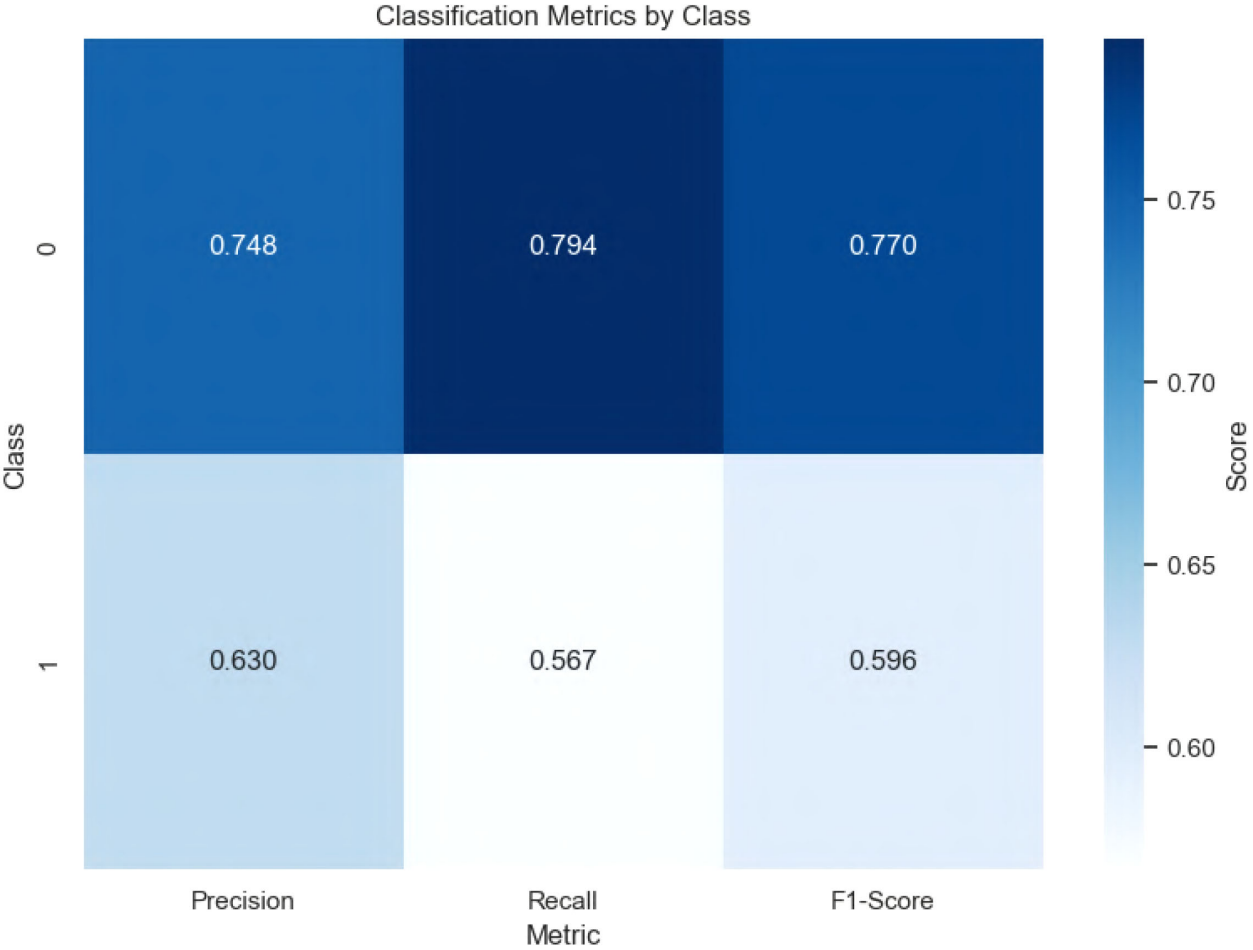
Heatmap of classification metrics (precision, recall, F1-score) for each class on the test set.

The XGBoost model demonstrated solid performance on the test set derived from an 80–20 train–test split of the dataset. The classification report, detailed in **Figure 3**, reveals the precision, recall, and F1-score for each class. For class 0 (e.g., “no ADHD”), the model achieved a precision of 0.748, recall of 0.794, and F1-score of 0.770. For class 1 (e.g., “ADHD”), the precision was 0.630, with a recall of 0.567 and an F1-score of 0.596. These metrics indicate that the model performs better at identifying non-ADHD cases, likely due to class imbalance or stronger predictive signals in the features associated with the absence of ADHD. The heatmap visualization highlights these differences, with darker shades representing higher scores, suggesting a need for further investigation into class-specific challenges.

### 4.2 Model performance validation

**Figure 4:**
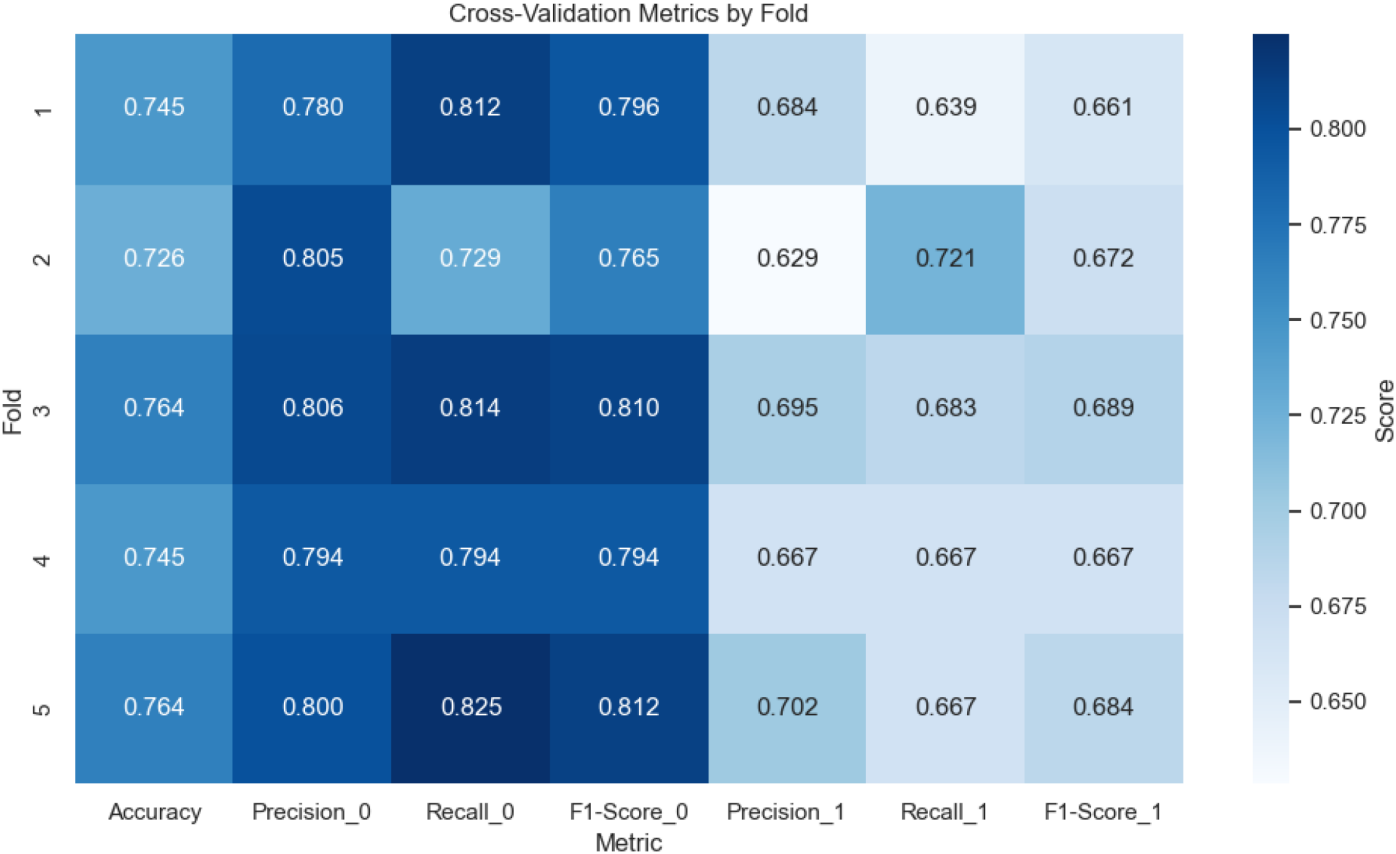
Heatmap of cross-validation metrics (accuracy, precision, recall, F1-score) by fold.

To assess the stability and generalizability of the model, a fivefold stratified cross-validation was performed. The results, presented in **Table 2** and visualized in **Figure 4**, show consistent performance across folds. The average accuracy across the five folds was 0.775, with a range from 0.745 to 0.764, indicating reliable predictive power. For class 0, the average precision, recall, and F1-score were 0.805, 0.814, and 0.810, respectively, while for class 1, these values were 0.695, 0.683, and 0.689. The heatmap in **Figure 4** uses a color gradient to emphasize fold-specific variations, with the darker blues indicating higher scores. The consistency across folds suggests that the model is not overly sensitive to the specific train–test split, reinforcing its robustness for this dataset.

**Table 2:** Cross-validation results showing accuracy and class-specific metrics for each fold.

Fold	Accuracy	Precision_0	Recall_0	F1-score_0	Precision_1	Recall_1	F1-score_1
1	0.745	0.780	0.812	0.796	0.684	0.639	0.661
2	0.726	0.805	0.729	0.765	0.629	0.721	0.672
3	0.764	0.806	0.814	0.810	0.695	0.683	0.689
4	0.745	0.794	0.794	0.794	0.667	0.667	0.667
5	0.764	0.800	0.825	0.812	0.702	0.667	0.684

### 4.3 Global feature importance

**Figure 5:**
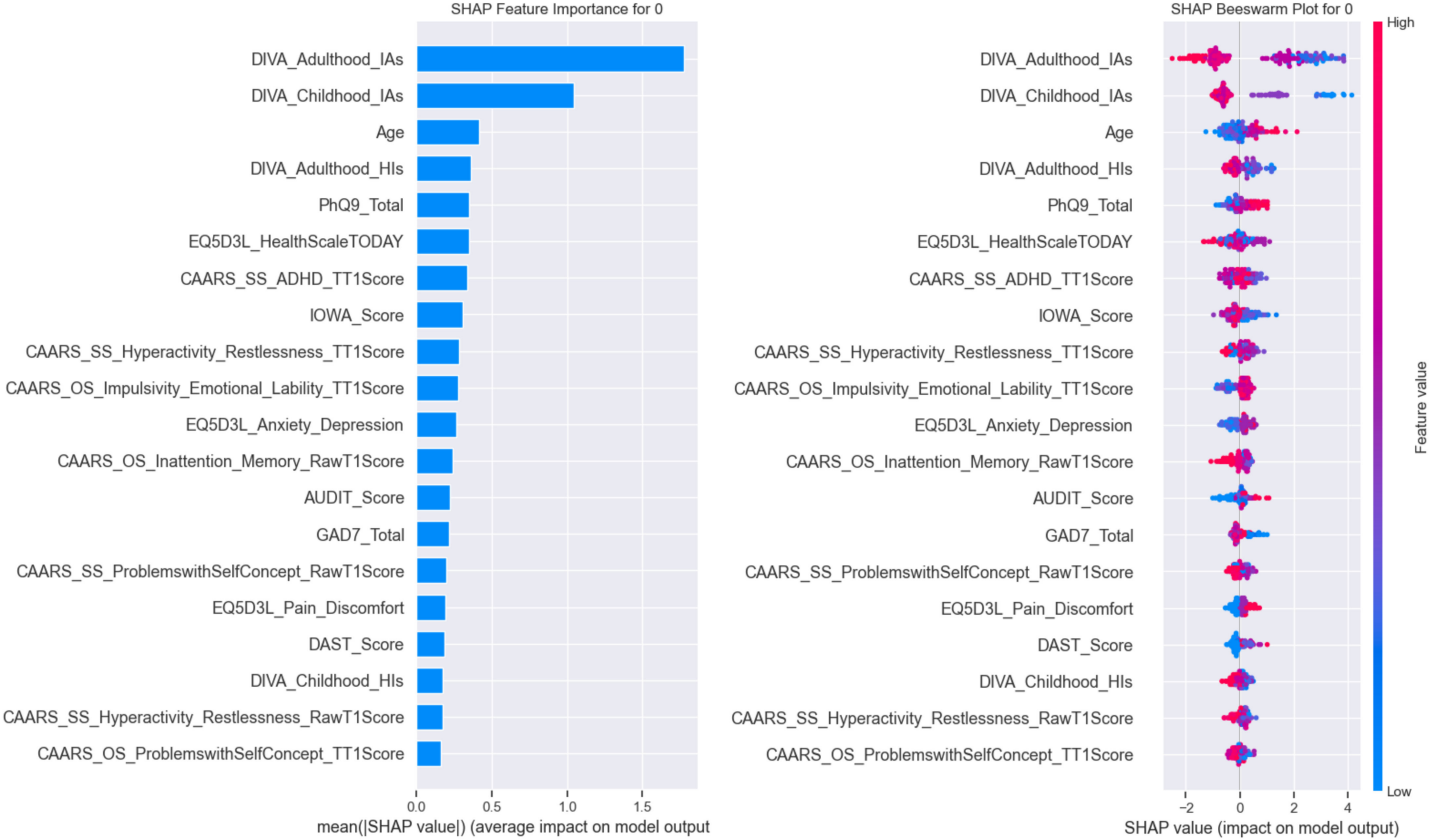
SHAP feature importance bar plot for non-ADHD (class 0).

**Figure 6:**
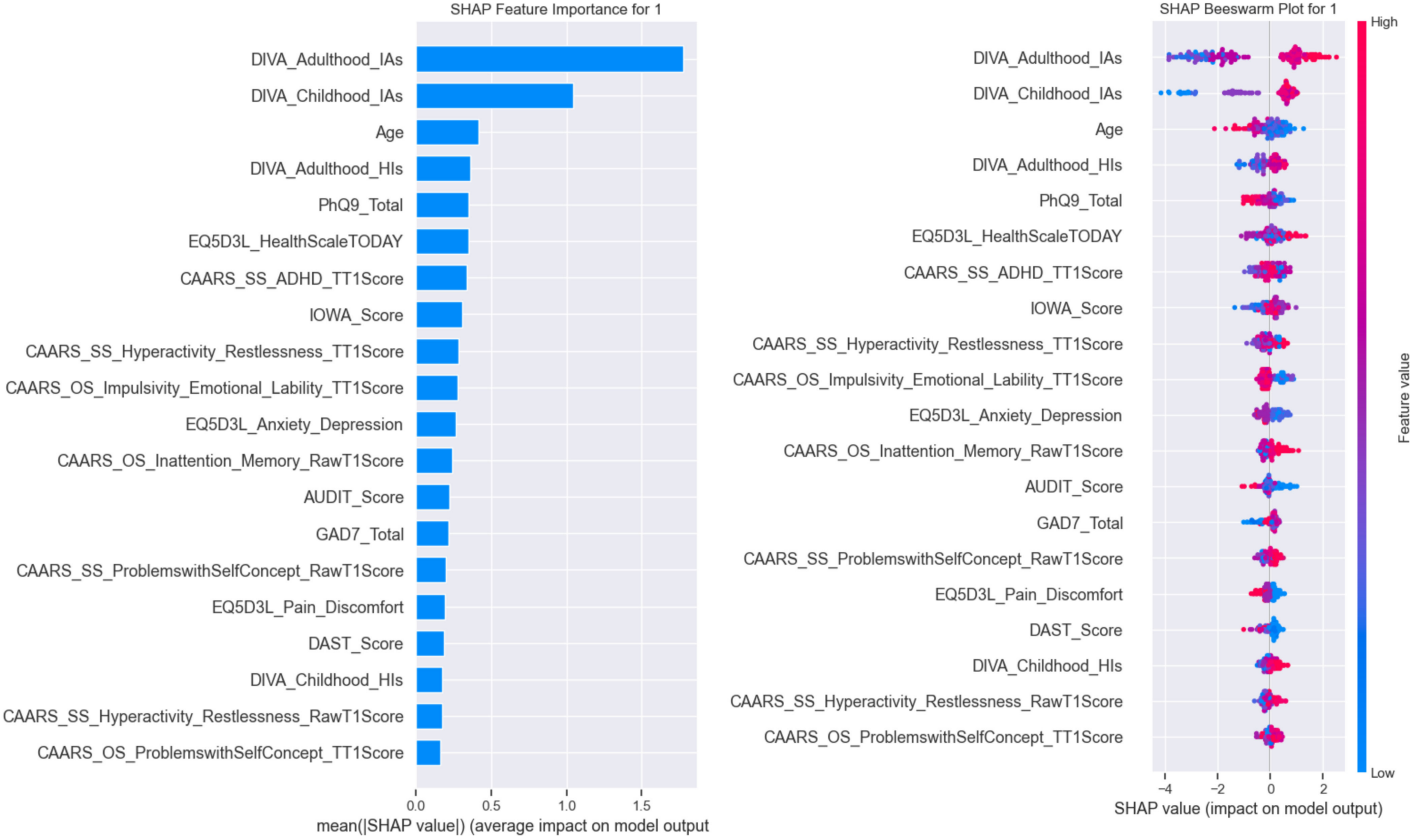
SHAP feature importance bar plot for ADHD (class 1).

Before delving into individual patient examples, we first present a high-level overview of how key features drive the model’s overall ADHD predictions. By aggregating SHAP values across the entire cohort, global plots identify the measures with the greatest average impact—revealing the relative importance of core inattentive symptoms, functional health scores, and comorbid mood and substance use indicators. These broad insights establish the primary axes along which the model separates ADHD from non-ADHD cases and set the stage for the detailed, case-level analyses that follow. The SHAP (SHapley Additive exPlanations) analysis provides a detailed breakdown of feature importance and interaction for each class, offering insights into which variables most influence the model’s predictions. **Figures 5** illustrates the feature importance and beeswarm plots for non-ADHD, while **Figures 6** presents the same for ADHD cases.

#### 4.3.1 Class-wise explainability for non-ADHD (class 0) cases

For non-ADHD, the bar plot in **Figure 5** highlights “DIVA_Adulthood_IAs” and “DIVA_Childhood_IAs” as the most impactful features, with mean SHAP values exceeding 1, suggesting that these variables strongly support the prediction of non-ADHD cases. The beeswarm plot in **Figure 5** shows that the high values of “DIVA_Adulthood_IAs” consistently increase the likelihood of class 0, indicating that adult ADHD symptom severity (as assessed by the DIVA tool) plays a critical role in ruling out ADHD. The beeswarm plot further reveals that while “Age” and “PhQ9_Total” have moderate impacts, their effects vary across individuals, with some high values pushing predictions toward class 0 and others toward ADHD (class 1), reflecting the complexity of these features.

#### 4.3.2 Class-wise explainability for ADHD (class 1) cases

For class 1 (ADHD), the bar plot in **Figure 6** again identifies “DIVA_Adulthood_IAs” as the top contributor, but with a slightly lower mean SHAP value compared with class 0, suggesting a bidirectional influence depending on the feature’s magnitude. The beeswarm plot in **Figure 6** indicates that low values of “DIVA_Adulthood_IAs” and “DIVA_Childhood_IAs” strongly drive predictions toward ADHD, aligning with clinical expectations that lower symptom severity in adulthood or childhood might indicate the absence of the condition. The beeswarm plot also shows that “AUDIT_Score” and “GAD7_Total” have scattered impacts, with some individuals exhibiting high anxiety or substance use scores that push predictions toward ADHD, highlighting potential comorbidities.

The differing importance of features across classes suggests that the model captures distinct patterns—for instance, the prominence of DIVA-related features across both classes underscores their diagnostic relevance, while the varying influence of “Age” and psychological scores (e.g., “PhQ9_Total,” “GAD7_Total”) points to age-related or mental health-related nuances in ADHD presentation. This interpretation aligns with clinical literature (61), where adult ADHD symptoms and comorbidities like anxiety or depression often complicate diagnosis.

#### 4.3.3 Class-wise interpretation of feature influence

The class-specific SHAP beeswarm analyses in **Figures 5**, **6** provide a clear understanding of how individual features influenced the likelihood of ADHD versus non-ADHD diagnoses. For non-ADHD cases (class 0), the SHAP importance and beeswarm plots in **Figure 5** identified that lower DIVA adulthood inattentiveness scores, childhood inattentiveness scores, and younger age significantly reduced ADHD diagnosis likelihood. Specifically, lower scores on these scales are strongly associated with negative ADHD diagnoses, highlighting their diagnostic specificity. Lower PHQ-9 total scores, indicating fewer depressive symptoms, also contributed substantially to non-ADHD classifications. The EQ-5D-3L Health Scale scores suggested better perceived overall health associated with non-ADHD outcomes.

Conversely, ADHD diagnoses (class 1) displayed a mirrored yet distinct profile in **Figure 6**. Higher DIVA adulthood and childhood inattentiveness scores prominently increased ADHD diagnosis likelihood. Elevated age also moderately contributed, reflecting adult diagnosis and potential late recognition of symptoms. Increased PHQ-9 total scores indicating higher depressive symptom severity were also relevant for predicting ADHD presence. Similarly, reduced EQ-5D-3L Health Scale scores suggested poorer perceived health among ADHD cases, aligning with clinical observations of compromised life quality. Further SHAP value distributions emphasized the nuanced impacts of ADHD-specific scales such as CAARS hyperactivity—restlessness and impulsivity—emotional lability. Elevated scores on these measures distinctly differentiated ADHD cases from non-ADHD ones. The AUDIT and DAST substance use scales likewise demonstrated moderate but meaningful influences, with higher substance use scores mildly elevating ADHD prediction probabilities. Collectively, these class-wise SHAP interpretations highlighted critical differences in the influence of features between ADHD and non-ADHD cases, providing nuanced insights crucial for targeted clinical assessments and individualized intervention strategies.

#### 4.3.4 Class-wise SHAP dependence analysis

In **Supplementary Figure S5**, SHAP dependence plots reveal how individual feature values influence ADHD prediction differently across classes. For non-ADHD cases (class 0), higher age is associated with increasingly negative SHAP values, indicating that older age strongly reduces the likelihood of an ADHD diagnosis. Substance use measures (AUDIT, DAST) show a mild downward trend: low AUDIT scores (*<*5) yield near-zero SHAP contributions, whereas very high scores (*>*21) slightly push toward non-ADHD. Depressive symptom severity (PHQ-9) exhibits a weak negative association with ADHD probability in class 0, suggesting that mild to moderate depression alone does not indicate ADHD. Anxiety scores (GAD-7) remain near zero for most non-ADHD individuals, with a slight uptick only at the highest anxiety levels. Core ADHD symptom scores (CAARS SS ADHD RawT1) have minimal impact in the non-ADHD group, underscoring their specificity to positive predictions.

In ADHD-positive cases (class 1), the dependence plots that can be seen in **Supplementary Figure S6** invert these relationships. Elevated PHQ-9 scores correlate with strongly positive SHAP values, indicating that higher depressive symptomatology magnifies the model’s confidence in ADHD. Similarly, AUDIT scores below 10 contribute modestly positive SHAP values, while scores above 20 begin to plateau, suggesting that moderate alcohol use compounds ADHD risk up to a threshold. GAD-7 displays a non-monotonic “U-shaped” effect: mild anxiety (0–5) slightly lowers ADHD probability, whereas moderate to severe anxiety (10–20) raises it. Notably, CAARS SS ADHD RawT1 exhibits a steep positive slope: raw scores above 25 sharply increase SHAP contributions, pinpointing a critical severity threshold. Age shows diminished positive SHAP values for older adults, reinforcing that ADHD symptom expression is most pronounced and diagnostically clear in younger patients.

#### 4.3.5 SHAP interaction effects (class-wise)

Interaction analyses capture synergistic feature effects that are not evident in univariate dependence. In **Supplementary Figure S7** for non-ADHD predictions, CAARS SS ADHD RawT1 × Age interactions remain near zero, indicating that inattention severity does not meaningfully interact with age to influence non-ADHD classification. Conversely, the PHQ-9 × GAD-7 plot shows slight negative interaction values at combined high anxiety and depression, suggesting that co-occurring mood symptoms reinforce non-ADHD predictions when ADHD symptoms are minimal. CAARS × AUDIT interactions hover around zero, implying that substance use does not substantially alter the low ADHD risk of non-ADHD individuals.

In **Supplementary Figure S8** ADHD positives, significant interactions emerge. CAARS SS ADHD RawT1 × Age shows that high inattention scores in younger adults yield stronger positive SHAP interactions than identical scores in older adults. This underscores age-sensitive symptom salience. The PHQ-9 × GAD-7 interaction is pronounced: individuals with both high depression (*>*20) and high anxiety (*>*15) see a synergistic boost in ADHD probability (SHAP interaction >0.1), reflecting compounded mood-symptom effects. CAARS × AUDIT interactions become positive for raw scores above 30 and AUDIT scores between 10 and 20, indicating that when core ADHD severity and moderate alcohol use coincide, ADHD predictions intensify.

#### 4.3.6 Partial dependence insights (class-wise)

In **Supplementary Figure S9**, partial dependence plots synthesize global trends. For non-ADHD, PHQ-9 and GAD-7 partial plots show flat or slightly negative SHAP values across the score range, confirming that mood distress alone does not drive ADHD classification. AUDIT and DAST partial curves remain near zero, further validating minimal substance use influence. Age partial dependence declines monotonically, reinforcing its protective effect. CAARS SS ADHD RawT1 partial dependence is flat for low scores (*<*20) and then dips slightly negative before rising modestly, showing that only extreme scores impact non-ADHD predictions.

In ADHD-positive partial plots of **Supplementary Figure S10**, PHQ-9 and GAD-7 curves slope upward, highlighting that increasing mood symptoms systematically raise ADHD probability. AUDIT partial dependence peaks around scores of 10–15, suggesting an optimal substance use range where ADHD risk is maximized before plateauing. DAST remains relatively flat, indicating minimal average effect. Age partial plots decline, aligning with dependence findings. CAARS SS ADHD RawT1 exhibits the steepest rise among all features, with a clear inflection at 25, solidifying its role as the most sensitive continuous predictor.

### 4.4 Case-level explanation studies

To illustrate how our model reasons about individual patients, we examine two contrasting real-world scenarios: a correctly classified non-ADHD case and an ADHD case that the model misclassifies. For each patient, we traverse the SHAP decision plot showing how features accumulate from the base risk to the final prediction and then unpack the contributions with waterfall charts, heatmaps, and force plots. This granular analysis reveals precisely which clinical measurements tip the balance toward or away from an ADHD diagnosis, offering practitioners clear, case-specific insights into model behavior.

#### 4.4.1 Local explanation for non-ADHD cases

**Figure 7:**
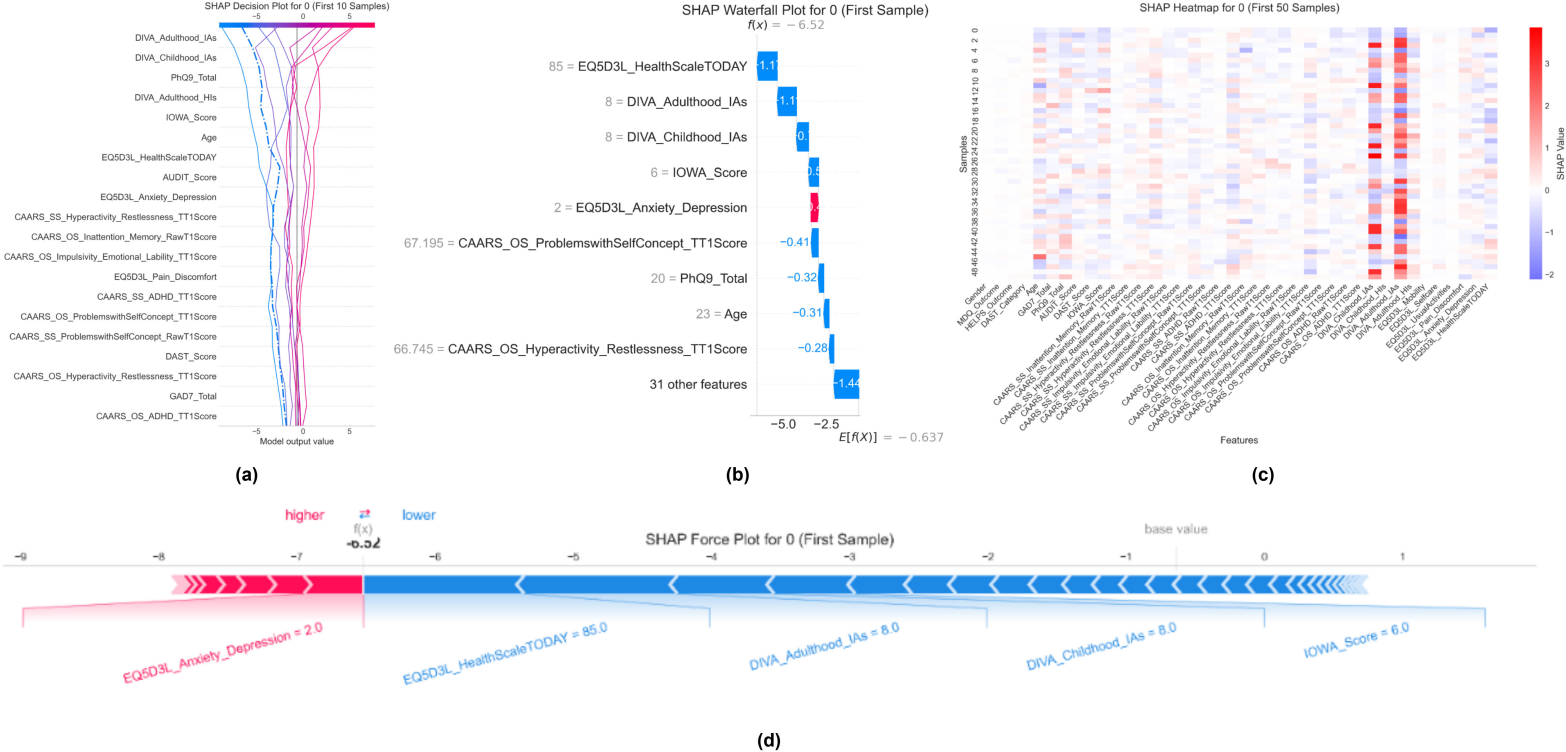
Local SHAP explanations for non-ADHD predictions (class 0). **(a)** SHAP decision plot for 10 non-ADHD samples, showing cumulative feature impacts. **(b)** Waterfall plot for the first non-ADHD case, detailing positive and negative feature contributions. **(c)** SHAP heatmap for the first 50 non-ADHD samples, illustrating feature heterogeneity. **(d)** SHAP force plot for the first non-ADHD case, mapping feature values to their impact.

In **Figure 7**, the SHAP decision plot for 10 non-ADHD samples is shown. **Figure 7** illustrates how individual feature contributions accumulate from the base value toward each patient’s final log-odds prediction. Across these cases, high values of EQ-5D-3L HealthScaleTODAY (better perceived health) consistently produce negative SHAP pushes (toward non-ADHD), while DIVA inattentiveness scores register minimal or negative impacts. As to the waterfall plot for the first sample, **Figure 7** quantifies these effects: strong negative contributions from EQ-5D-3L Anxiety/Depression (–1.1) and Pain/Discomfort (–1.0) outweigh small positive pushes from DIVA and IOWA scores, yielding an overall negative shift (from base = –0.64 to f(x) = –6.52). The SHAP heatmap in **Figure 7** for the first 50 non-ADHD cases confirms this pattern at scale: cooler blue bands appear under quality-of-life and mood features, whereas core ADHD scales show near-neutral contributions. Finally, the force plot in **Figure 7** visually emphasizes that protective factors good health scores and low depressive symptoms dominate the explanation, demonstrating how clinicians might recognize functional well-being as a key indicator of non-ADHD status.

#### 4.4.2 Local explanation for misclassified ADHD cases

**Figure 8:**
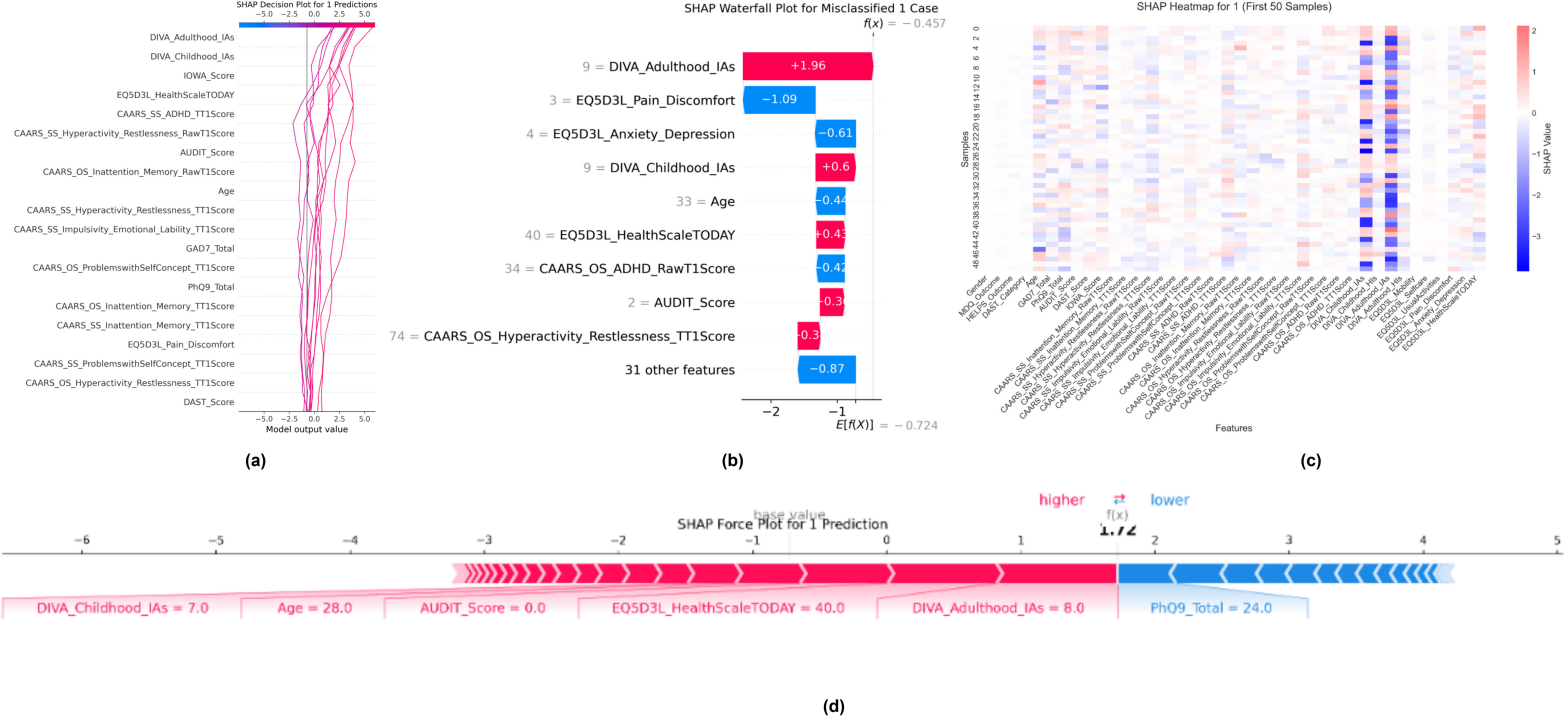
Local SHAP explanations for a misclassified ADHD prediction (class 1). **(a)** SHAP decision plot for 10 ADHD samples. **(b)** Waterfall plot for the misclassified ADHD case, showing how quality-of-life factors outweighed core symptom signals. **(c)** SHAP heatmap for the first 50 ADHD samples. **(d)** SHAP force plot for the misclassified ADHD case.

In contrast, the decision plot in **Figure 8** is for ADHD samples. **Figure 8** shows predominantly positive SHAP trajectories: high DIVA_Adulthood_IAs and DIVA_Childhood_IAs values drive predictions upward, yet in the misclassified case highlighted, the waterfall plot (**Figure 8**) reveals why the model under-calls ADHD: although DIVA inattentiveness contributes +1.96 and +0.60, substantial negative pulls from EQ-5D-3L Pain/Discomfort (–1.09) and Anxiety/Depression (–0.61) flip the net effect to –0.46, below the decision threshold. The corresponding heatmap in **Figure 8** for the first 50 ADHD-labeled patients shows this tension: red bands under DIVA columns contrast sharply with blue bands under quality-of-life, illustrating that exceptional well-being or low comorbid distress can counteract strong symptom signals. The force plot (**Figure 8**) for the misclassified individual underscores this dynamic, as positive pushes from DIVA scores (red) are almost entirely negated by negative pulls from quality-of-life and mood measures (blue). Clinically, these visualizations warn that robust functional health may mask severe inattentive symptoms, suggesting the need for deeper probing when core ADHD scales conflict with self-reported well-being.

### 4.5 Comparative analysis of EDA and SHAP

A central goal of our study was to analyze classical exploratory data analysis (EDA) with SHAP-based explainability to ensure that the model insights reflect genuine clinical patterns (“statistical ground truthing”) rather than artefacts of the algorithm. Early EDA steps characterized univariate distributions, bivariate associations, age histograms, and proportions that highlighted the concentration of ADHD cases among younger adults; *χ*^2^ and Welch’s *t*-tests confirmed that core symptom scales (CAARS, DIVA) and quality-of-life domains (EQ-5D-3L pain/discomfort, anxiety/depression) are statistically separate diagnostic groups, and pairwise Pearson correlations exposed multicollinearity clusters and ranked feature diagnosis linear relationships. These EDA findings established the foundational “signatures” of ADHD in our cohort and guided feature engineering choices. When we applied SHAP to the XGBoost model, global importance rankings and summary plots mirrored these EDA signals: DIVA inattentiveness and CAARS ADHD index scores emerged as the dominant predictors, consistent with their high t-statistics and *χ*^2^ effect sizes. Moreover, SHAP uncovered non-linear dependencies such as the inflection at CAARS raw ≈ 25 in partial dependence that EDA’s linear tests could not detect, yet critically, the features flagged by SHAP all corresponded to those that EDA had identified as statistically significant, validating the model’s internal reasoning and guarding against “Clever Hans” effects (34).

EDA also hinted at interactions: pairplots suggested that depressive and anxiety scales might modulate symptom severity effects, but the patterns were noisy. SHAP interaction plots precisely quantified these synergies showing, for example, that high PHQ-9 and GAD-7 jointly amplify the ADHD signal (interaction SHAP >0.1) and that age moderates CAARS inattention impacts in younger adults. These clear, model-derived interaction effects both confirm EDA’s qualitative suggestions and transform them into actionable insights for personalized assessment. By systematically cross-referencing SHAP attributions with EDA’s statistical benchmarks, we establish a transparent explainability framework; each feature contribution can be traced back to EDA-verified group differences, ensuring that clinicians can trust both the model’s accuracy and its rationale. This “transparent XAI with statistical ground truthing” not only bolsters confidence in our ADHD classifier but also provides a reproducible template for interpretable machine learning applications across mental health and beyond.

## 5 Discussion

This study demonstrates that explainable artificial intelligence provides a robust framework for understanding adult ADHD diagnosis through a systematic analysis of symptom interactions, comorbidities, and demographic factors. The XGBoost model achieved 82% accuracy while offering transparent insights into diagnostic decision-making processes. The integration of SHAP analysis with traditional statistical methods revealed complex, non-linear relationships that extend the current understanding of adult ADHD presentation. These findings have significant implications for clinical practice, theoretical conceptualization, healthcare delivery, and future research directions in adult neurodevelopmental disorders. ADHD-specific rating scales and tools emerged as dominant predictors, with CAARS and DIVA measures demonstrating the strongest influence on diagnostic outcomes. The non-linear relationship between these measures and diagnosis, particularly the threshold effect at CAARS scores of 25, has direct implications for clinical assessment protocols. This finding suggests that current linear interpretations of symptom severity may oversimplify the diagnostic process. Clinicians might benefit from adopting threshold-based approaches that recognize qualitative shifts in diagnostic probability at specific severity levels rather than assuming gradual linear increases. The moderating effect of depression on ADHD symptom interpretation carries significant implications for differential diagnosis. When PHQ-9 scores exceeded 18, attention problems became less distinctive for ADHD, indicating that standard ADHD assessments may lose specificity in the context of significant mood disturbance. This quantified interaction suggests that clinical services should consider sequential or hierarchical assessment strategies, potentially addressing mood symptoms before finalizing ADHD diagnoses in cases with significant depressive features. The finding also implies that treatment planning might require integrated approaches addressing both conditions simultaneously rather than treating them as independent disorders. Age-related variations in symptom expression and diagnostic weight have implications for service organization and assessment processes. The declining ADHD probability after age 40 and the differential diagnostic value of identical symptoms across age groups suggest that adult ADHD services require age-stratified assessment criteria. This challenges the current practice of applying uniform diagnostic thresholds across the adult lifespan. Healthcare systems might need to develop separate pathways or adjusted criteria for different age cohorts, recognizing that ADHD presentation in a 25-year-old differs qualitatively from that in a 50-year-old, even when symptom scores appear similar.

The discovery that high quality-of-life scores can override symptom signals has significant implications for understanding missed diagnoses and delayed recognition of ADHD in high-functioning individuals. This computational finding validates clinical observations that successful compensation strategies can obscure underlying neurodevelopmental differences. The implications extend to several domains. Screening protocols might need revision to include questions about the effort required to maintain function rather than function alone. Clinicians should consider the cognitive load and energy expenditure behind apparent success, particularly in individuals with high educational or occupational achievement who present with late-onset concerns. This phenomenon reflects the model’s appropriate alignment with DSM-5 diagnostic criteria, which require functional impairment for diagnosis. However, the finding highlights important considerations about how impairment is assessed and recognized in clinical practice. The model’s behavior—reducing ADHD probability when quality-of-life appears high—correctly applies current diagnostic standards while revealing potential limitations in how functional impairment is measured through standard quality-of-life instruments. The findings suggest that healthcare services might benefit from developing more nuanced approaches to assessing impairment that capture hidden costs of maintaining function. Rather than reconsidering whether impairment should be required for diagnosis, which would contradict established criteria, services could refine how impairment is identified and documented. This includes recognizing that impairment in “quality of functioning” (as specified in DSM-5) may manifest as excessive effort, chronic exhaustion, or secondary anxiety while maintaining an apparently adequate performance. The clinical implications point toward expanding assessment methods to capture domains of impairment not reflected in standard quality-of-life measures. Individuals who maintain function through unsustainable compensatory strategies may still meet the diagnostic criteria if assessment tools adequately capture the reduced quality of their functioning. This suggests that comprehensive assessment should explore not only current observable function but also the psychological and physical cost of maintaining that function, historical periods of clearer impairment, and impairment in domains that standard measures may overlook. The model’s support for dimensional rather than categorical conceptualization of ADHD has far-reaching theoretical implications. The observed overlap between ADHD, anxiety, and depression challenges traditional diagnostic boundaries and suggests that these conditions share underlying mechanisms related to executive and emotional regulation. This dimensional view implies that research should focus on identifying common neurobiological pathways and environmental factors that influence expression across these related conditions rather than seeking discrete etiologies for each disorder. The debate about which approach to use has been longstanding with benefits reported to each (62).

The trans-diagnostic patterns identified through SHAP analysis support emerging frameworks that organize psychopathology along functional dimensions rather than traditional diagnostic categories. This is similar to what is found in personality (63). This has implications for how research funding is allocated, how clinical trials are designed, and how treatment responses are measured. Studies might benefit from recruiting participants based on dimensional symptom profiles rather than categorical diagnoses, potentially revealing treatment effects that current diagnostic-based approaches miss. The quantification of symptom interactions through machine learning provides an empirical foundation for hierarchical models of psychopathology. The finding that depression moderates the expression and diagnostic value of ADHD symptoms suggests that these conditions exist in a hierarchical relationship (64) where mood disturbance can subsume or alter the presentation of attention problems. This implies that biological research should investigate shared neural circuits and genetic factors while acknowledging that expression may vary depending on which system is primarily affected. The transparent AI framework offers potential solutions to current challenges in adult ADHD services, including long waiting lists, diagnostic inconsistency, and resource allocation. The model could function as a triage tool, identifying cases requiring urgent specialist assessment versus those suitable for less intensive pathways. This stratified approach could improve service efficiency while ensuring that complex cases receive appropriate expert attention. The ability to quantify diagnostic probability based on routine screening measures has implications for workforce planning and training. Non-specialist staff could administer initial assessments with AI-supported interpretation, reserving specialist time for complex cases where clinical judgment becomes essential. This does not replace clinical expertise but redistributes it more effectively across the healthcare system. Primary care providers might use the framework to identify potential ADHD cases for referral, reducing the burden of inappropriate referrals while ensuring that valid cases reach specialist services. The model’s transparency facilitates quality assurance and service standardization. By making diagnostic reasoning explicit, services can identify sources of variability between clinicians and sites, developing targeted training to address inconsistencies. The framework could support newly qualified staff by providing decision support that explains its reasoning, accelerating the development of clinical pattern recognition skills. The implications for individuals seeking ADHD assessment are substantial. The framework could reduce diagnostic uncertainty by providing clear explanations for diagnostic decisions, helping patients understand why they do or do not meet criteria for ADHD. This transparency might improve acceptance of diagnostic outcomes and engagement with recommended interventions. Patients could see how different factors contribute to their presentation, facilitating more personalized discussions about treatment options.

The identification of symptom interactions and effects of coping strategies validates the experiences of many adults who struggle to articulate why they suspect ADHD despite maintaining apparent function. The framework provides language and evidence for these subjective experiences, potentially reducing the dismissal of concerns based solely on functional achievements. This could lead to earlier identification and intervention for individuals who might otherwise struggle for years before receiving appropriate support. The age-related findings have particular relevance for older adults seeking assessment, who often report feeling dismissed because their presentation differs from younger adults or childhood ADHD stereotypes. The empirical demonstration of age-related symptom evolution supports the need for age-appropriate assessment and challenges assumptions that ADHD necessarily diminishes with age (65). The successful integration of explainable AI with clinical data establishes a methodological template to address complex psychiatric diagnoses. The dual validation approach, combining machine learning with traditional statistics, provides a robust framework to ensure that computational models reflect genuine clinical phenomena rather than algorithmic artefacts. This methodology could extend to other psychiatric conditions where multiple factors interact to influence diagnosis and treatment outcomes. The SHAP framework’s ability to visualize feature interactions offers new ways to communicate complex clinical concepts to both professionals and patients. These visualizations could enhance clinical training by making abstract diagnostic reasoning concrete and observable. Medical education might incorporate such tools to teach pattern recognition and clinical reasoning skills more effectively than traditional didactic methods allow. Future developments could incorporate longitudinal data to model symptom trajectories and treatment responses over time. The framework could evolve from supporting single-timepoint diagnosis to predicting clinical course and treatment outcomes. Integration with genetic, neuroimaging, and environmental data could further enhance the model’s sophistication while maintaining interpretability through the SHAP framework. The findings contribute to ongoing debates about psychiatric classification systems. The dimensional patterns and symptom interactions identified challenge the validity of discrete diagnostic categories, supporting calls for dimensional approaches in future diagnostic manuals. The quantification of how comorbidities influence symptom expression provides empirical support for hierarchical or network models of psychopathology that recognize the interdependence of mental health conditions. This work demonstrates that complex psychiatric phenomena need not remain opaque to systematic analysis. By combining clinical expertise with transparent computational methods, the field can move beyond reliance on subjective interpretation toward more objective, reproducible diagnostic processes while maintaining the nuanced understanding that characterizes good clinical practice. The framework shows that technological advancement in psychiatry need not come at the cost of clinical interpretability or patient-centered care. The integration of explainable AI into adult ADHD assessment represents a significant step toward precision psychiatry, where diagnosis and treatment are tailored to individual presentations rather than categorical assumptions. As healthcare systems face increasing demand for adult ADHD services, such frameworks offer pathways to improve efficiency, consistency, and outcomes while maintaining the human elements essential to psychiatric care. The challenge moving forward lies not in choosing between clinical judgment and computational analysis but in optimizing their integration to serve patients more effectively.

## 6 Implications

The present study bridges a critical gap by demonstrating that an interpretable XGBoost model, guided by SHAP analyses and traditional EDA, can robustly predict adult ADHD diagnoses using routinely collected clinical data. By identifying core symptom scales (CAARS, DIVA), mood assessments (PHQ-9, GAD-7), substance use screens (AUDIT, DAST), and quality-of-life indices (EQ-5D-3L) as principal drivers of diagnostic outcomes, our work expands the empirical foundation for data-driven psychiatric evaluation. Integrating modal measures within a transparent framework ensures that predictive insights align with established clinical knowledge while revealing non-linear and interaction effects previously unquantified.

### 6.1 Theoretical implications

This study advances theoretical models of ADHD by reframing symptom severity and comorbidity as interdependent constructs rather than isolated predictors. The discovery of meaningful interactions such as elevated depressive symptoms magnifying the impact of ADHD-specific scales suggests that ADHD manifests through a network of overlapping features rather than a single latent dimension. These findings challenge unitary trait models and support a multifactorial, dimensional perspective in which mood, cognition, and functional impairment dynamically interact. Moreover, identifying age and gender as moderating variables provides a nuanced lens for theory building, indicating that developmental and sociocultural factors shape symptom expression and should be integrated into future conceptualizations.

### 6.2 Practical implications

Clinicians can leverage the identified SHAP-derived thresholds (e.g., CAARS Raw score >30; PHQ-9 total >18) as objective markers to streamline screening and triage decisions. Embedding these cutoffs within digital assessment platforms could standardize initial evaluations, reduce diagnostic variability, and prioritize resource allocation for high-risk patients. The model’s capacity to generate individualized explanations fosters shared decision-making by illustrating how each measure influences diagnostic probability, thereby enhancing practitioner–patient communication and trust. Additionally, incorporating substance use assessments alongside core ADHD scales underscores the necessity of comprehensive intake procedures, encouraging integrated care pathways that simultaneously address mood, cognition, and lifestyle factors.

### 6.3 Limitations

While our retrospective design limits immediate generalizability, the methodology’s transparency and reproducibility support rapid validation in diverse settings. The clinical measures used here are widely implemented, making the approach adaptable to different health systems. Future research should explore prospective cohorts, incorporate medication adherence and socio-economic indicators, and test the integration of real-time explanatory feedback. In summary, this study demonstrates that combining explainable ML techniques with rich clinical data produces actionable insights for ADHD diagnosis and exemplifies a scalable blueprint for transparent, AI-assisted healthcare interventions. By harmonizing statistical rigor with clinical interpretability, we move toward a new paradigm of personalized, data-informed mental health care. Although the dataset spans 2019–2024, each record represents a single assessment per individual, making the structure cross-sectional rather than longitudinal. Temporal validation—training on earlier years and testing on later ones—was not applied in this initial phase to preserve sample size. As data collection continues, this analysis will be conducted to evaluate potential temporal drift and confirm model robustness over time.

## 7 Conclusion

This study demonstrates that combining an XGBoost classifier with rigorous SHAP-based interpretability and traditional exploratory analyses can effectively distinguish ADHD from non-ADHD presentations using a comprehensive set of clinical, demographic, substance use, and quality-of-life measures. Key predictors such as CAARS ADHD symptom severity, DIVA inattentiveness scores, and PHQ-9 depressive symptoms emerged consistently across methods, achieving 78% classification accuracy and 0.82 AUC-ROC. SHAP analyses further revealed nuanced, non-linear interactions between psychological distress, age, and substance use, offering granular insights beyond population-level trends. The findings hold significant implications for clinical practice by providing transparent, data-driven thresholds (e.g., CAARS Raw T1 score >30, PHQ-9 *>*18) and explaining individual diagnostic predictions. This approach advances diagnostic precision and fosters clinician trust in ML applications by aligning model behavior with established symptom profiles and comorbidity patterns. Moreover, identifying age- and gender-specific interaction effects addresses critical equity considerations, ensuring tailored assessment strategies across diverse patient subgroups.

Beyond ADHD, the integrative framework underscores the value of embedding explainable AI within psychiatric diagnostics and other mental health domains. By validating model explanations against EDA outcomes, we set a precedent for robust methodological cross-checks that enhance reliability. Health systems can leverage these insights to develop decision support tools that augment clinician capacity, streamline referrals, and ultimately improve patient outcomes. Future research should expand validation to multi-center cohorts, incorporate longitudinal data to track feature dynamics over treatment, and explore additional contextual factors such as medication adherence and socioeconomic status. Integrating real-time SHAP feedback within electronic health records could further personalize care pathways. Collectively, this work lays the groundwork for transparent, scalable, and equitable AI-driven diagnostic aids in mental health care.

## Data Availability

The data analyzed in this study is subject to the following licenses/restrictions: The dataset used in this study consists of anonymized clinical assessments collected as part of routine service delivery at South West Yorkshire Partnership NHS Foundation Trust (SWYPFT). Access to the raw data is restricted under NHS governance and UK data protection regulations, and it cannot be shared publicly due to patient confidentiality. The data were accessed with approval from the SWYPFT Research and Development Department, under classification as a service improvement activity, with Caldicott Guardian oversight. Patients were informed that their data could be used for research purposes and were given the opportunity to opt out. Secondary use of the dataset is therefore limited to authorized researchers within the Trust or approved collaborators, subject to NHS ethical and governance procedures. Requests to access these datasets should be directed to G.Antoniou@leedsbeckett.ac.uk.

## Supplementary material

Supplementary Figure 1 Demographic distributions of ADHD and non-ADHD cases. **(a)** Male and female counts by diagnosis. **(b)** Age-by-diagnosis swarm plot showing individual patient ages. **(c)** Proportion of ADHD diagnoses within four age ranges. **(d)** Stacked bar chart of ADHD *vs*. non-ADHD counts by age range.

Supplementary Figure 2 Comparative distributions of categorical and continuous predictors by diagnostic group. **(a)** Bar charts for categorical variables: DAST substance use category, EQ-5D-3L quality-of-life dimensions (anxiety/depression, mobility, pain/discomfort, self-care, usual activities), gender, HELPS brain-injury screen outcome, and MDQ bipolar screen outcome. **(b)** Overlaid histograms and density plots for continuous variables: age, AUDIT alcohol-use score, CAARS SS ADHD raw total score, DAST score, GAD-7 anxiety score, and PHQ-9 depression score, stratified by ADHD (orange) and non-ADHD (blue) diagnoses.

Supplementary Figure 3 Correlation analyses among top features: **(a)** Pearson correlation heatmap for the 20 most influential predictors; **(b)** pairplot of selected continuous variables with histograms and scatterplots colored by diagnosis; and **(c)** bar chart of Pearson correlation coefficients between each top feature and ADHD diagnosis ordered by absolute value.

Supplementary Figure 4 Statistical comparisons of diagnostic groups: **(a)** Cramér’s *V* from chi-square tests for categorical features versus ADHD diagnosis. **(b)** Welch’s *t*-statistics comparing numerical features for ADHD *vs*. non-ADHD (left) and for gender differences (right), with significant (*p <* 0.05) values shaded.

Supplementary Figure 5 SHAP dependence plots for non-ADHD (class 0): **(a)** age, **(b)** DAST score, **(c)** PHQ-9 total, **(d)** AUDIT score, **(e)** CAARS SS ADHD RawT1 score, and **(f)** GAD-7 total.

Supplementary Figure 6 SHAP dependence plots for ADHD (class 1): **(a)** GAD-7 total, **(b)** AUDIT score, **(c)** CAARS SS ADHD RawT1 score, **(d)** PHQ-9 total, and **(e)** age.

Supplementary Figure 7 SHAP interaction plots for non-ADHD (class 0): **(a)** CAARS × age, **(b)** PHQ-9 × GAD-7, and **(c)** CAARS × AUDIT.

Supplementary Figure 8 SHAP interaction plots for ADHD (class 1): **(a)** CAARS × age, **(b)** PHQ-9 × GAD-7, and **(c)** CAARS × AUDIT.

Supplementary Figure 9 SHAP partial dependence plots for non-ADHD (class 0): **(a)** PHQ-9 total, **(b)** AUDIT score, **(c)** DAST score, **(d)** GAD-7 total, **(e)** age, and **(f)** CAARS SS ADHD RawT1 score.

Supplementary Figure 10 SHAP partial dependence plots for ADHD (class 1): **(a)** PHQ-9 total, **(b)** AUDIT score, **(c)** DAST score, **(d)** GAD-7 total, **(e)** age, and **(f)** CAARS SS ADHD RawT1 score.
