# Supplementary material for: Integrating explainable AI with clinical features to enhance ADHD diagnostic understanding
Source: Front Psychiatry. 2025 Nov 26;16:1706216. doi: 10.3389/fpsyt.2025.1706216 (PMC12690393; doi:10.3389/fpsyt.2025.1706216)
Supplement: Supplementary Figure 1 — Demographic distributions of ADHD and non-ADHD cases. (a) Male and female counts by diagnosis. (b) Age-by-diagnosis swarm plot showing individual patient ages. (c) Proportion of ADHD diagnoses within four age ranges. (d) Stacked bar chart of ADHD vs. non-ADHD counts by age range. [file Presentation1.pdf]

Supplementary Material

Overview

This section provides additional figures, tables, and methodological details supporting the analyses presented in the main article. All supplementary figures are provided in high-resolution (600 dpi) format to ensure clarity for both print and online versions.

Equations

$$y_i = \text{LabelEncoder}(x_i), \tag{S1}$$

where x_i is the original categorical value and y_i is the encoded integer used by the model.

$$L(\hat{y}, y) = -\frac{1}{n}\sum_{i=1}^{n}\left[y_i \log(\hat{y}_i) + (1 - y_i)\log(1 - \hat{y}_i)\right] \tag{S2}$$

where $\hat{y}_i$ is the predicted probability of ADHD for patient i, y_i is the true diagnostic label (0 for non-ADHD, 1 for ADHD), and $n = 629$ represents the training set size used for model fitting.

$$f(x) = \phi_0 + \sum_{i=1}^{M}\phi_i, \tag{S3}$$

where ϕ_0 is the expected prediction over the training set and ϕ_i is the Shapley value for feature i.

Each ϕ_i reflects the average marginal contribution of feature i across all possible feature subsets $S \subseteq F \setminus \{i\}$:

$$\phi_i = \sum_{S \subseteq F \setminus \{i\}} \frac{|S|!\,(M - |S| - 1)!}{M!}\left[f_{S\cup\{i\}}(x) - f_S(x)\right]. \tag{S4}$$

This formulation ensures that the sum of all ϕ_i values (plus the base value ϕ_0) exactly equals the model prediction $f(x)$, satisfying the SHAP properties of *efficiency* (no loss of total contribution) and *consistency* (adding a more influential feature never decreases its contribution).

$$\phi_{i,j} = \sum_{S \subseteq F \setminus \{i,j\}} \frac{|S|!\,(M - |S| - 2)!}{2\,M!}\Big[f_{S\cup\{i,j\}}(x) - f_{S\cup\{i\}}(x) - f_{S\cup\{j\}}(x) + f_S(x)\Big], \tag{S5}$$

This equation quantifies the extra predictive lift (or suppression) jointly attributable to features i and j, beyond the sum of their individual contributions. By plotting $\phi_{i,j}$ values against feature i and coloring by feature j, the interaction effects can be visualised to reveal synergistic or antagonistic relationships between predictors.

Figures

- **Figure S1.** Demographic distributions of ADHD and non-ADHD cases. (a) Male and female counts by diagnosis; (b) Age-by-diagnosis swarm plot showing individual patient ages; (c) Proportion of ADHD diagnoses within four age ranges; (d) Stacked bar chart of ADHD vs. non-ADHD counts by age range.

- **Figure S2.** Comparative distributions of categorical and continuous predictors by diagnostic group. (a) Bar charts for categorical variables: DAST substance-use category, EQ-5D-3L quality-of-life dimensions (Anxiety/Depression, Mobility, Pain/Discomfort, Self-Care, Usual Activities), Gender, HELPS brain-injury screen outcome, and MDQ bipolar screen outcome; (b) Overlaid histograms and density plots for continuous variables: age, AUDIT alcohol-use score, CAARS SS ADHD raw total score, DAST score, GAD-7 anxiety score, and PHQ-9 depression score, stratified by ADHD (orange) and non-ADHD (blue) diagnoses.

- **Figure S3.** Correlation analyses among top features: (a) Pearson correlation heatmap for the 20 most influential predictors; (b) pairplot of selected continuous variables with histograms and scatterplots colored by diagnosis; (c) bar chart of Pearson correlation coefficients between each top feature and ADHD diagnosis, ordered by absolute value.

- **Figure S4.** Statistical comparisons of diagnostic groups: (a) Cramér's V from chi-square tests for categorical features versus ADHD diagnosis; (b) Welch's t-statistics comparing numerical features for ADHD vs. non-ADHD (left) and for gender differences (right), with significant ($p < 0.05$) values shaded.

- **Figure S5.** SHAP dependence plots for non-ADHD (class 0): (a) Age; (b) DAST Score; (c) PHQ-9 Total; (d) AUDIT Score; (e) CAARS SS ADHD RawT1 Score; (f) GAD-7 Total.

- **Figure S6.** SHAP dependence plots for ADHD (class 1): (a) GAD-7 Total; (b) AUDIT Score; (c) CAARS SS ADHD RawT1 Score; (d) PHQ-9 Total; (e) Age.

- **Figure S7.** SHAP interaction plots for non-ADHD (class 0): (a) CAARS × Age; (b) PHQ-9 × GAD-7; (c) CAARS × AUDIT.

- **Figure S8.** SHAP interaction plots for ADHD (class 1): (a) CAARS × Age; (b) PHQ-9 × GAD-7; (c) CAARS × AUDIT.

- **Figure S9.** SHAP partial dependence plots for non-ADHD (class 0): (a) PHQ-9 Total; (b) AUDIT Score; (c) DAST Score; (d) GAD-7 Total; (e) Age; (f) CAARS SS ADHD RawT1 Score.

- **Figure S10.** SHAP partial dependence plots for ADHD (class 1): (a) PHQ-9 Total; (b) AUDIT Score; (c) DAST Score; (d) GAD-7 Total; (e) Age; (f) CAARS SS ADHD RawT1 Score.

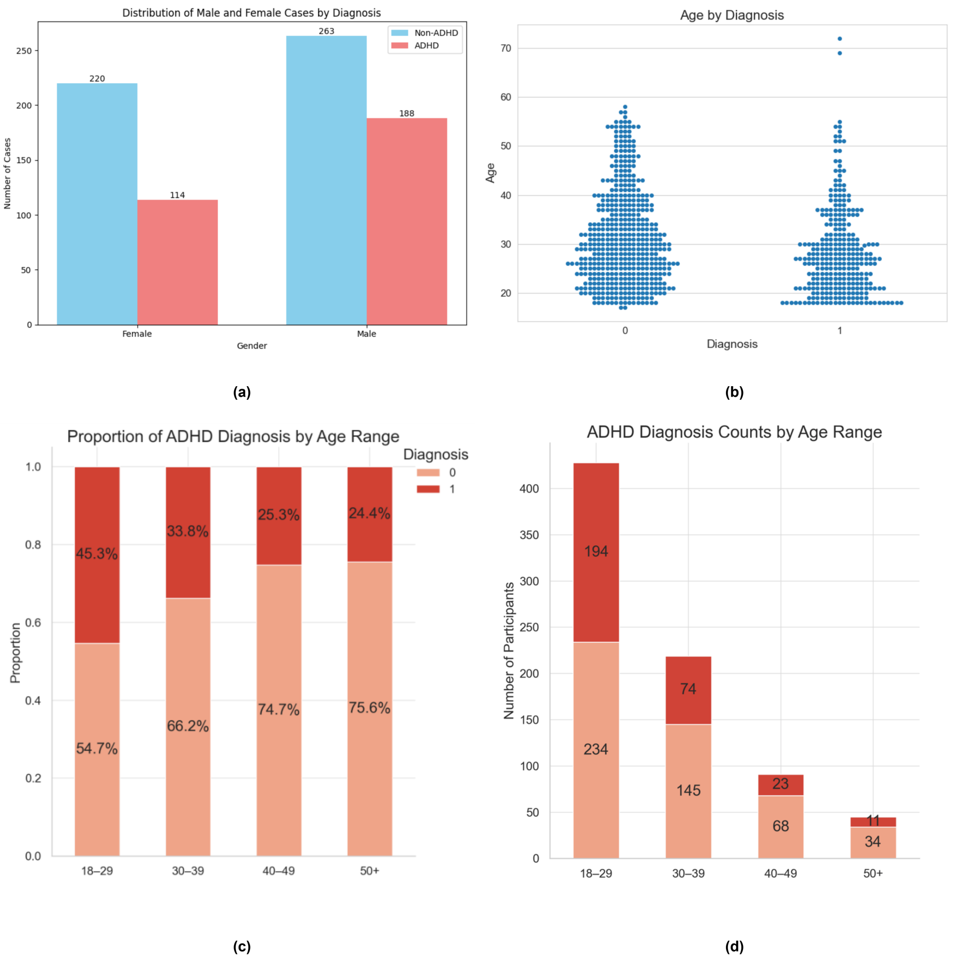

Figure S1: Demographic distributions of ADHD and non-ADHD cases. (a) Male and female counts by diagnosis; (b) Age-by-diagnosis swarm plot showing individual patient ages; (c) Proportion of ADHD diagnoses within four age ranges; (d) Stacked bar chart of ADHD vs. non-ADHD counts by age range.

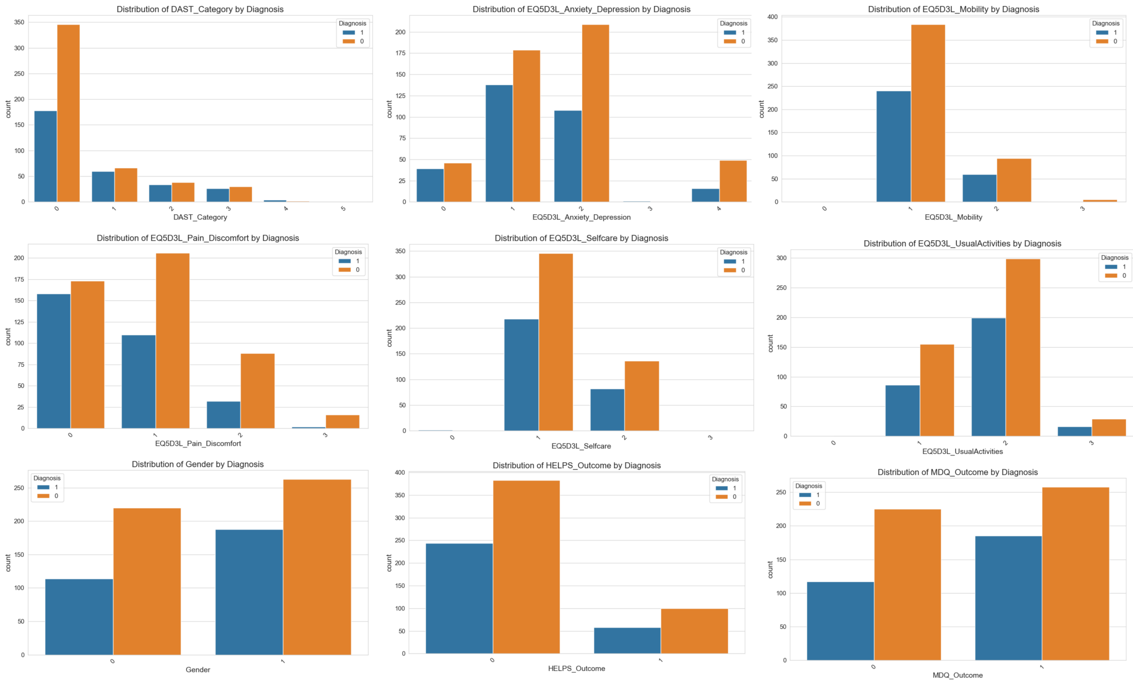

(a)

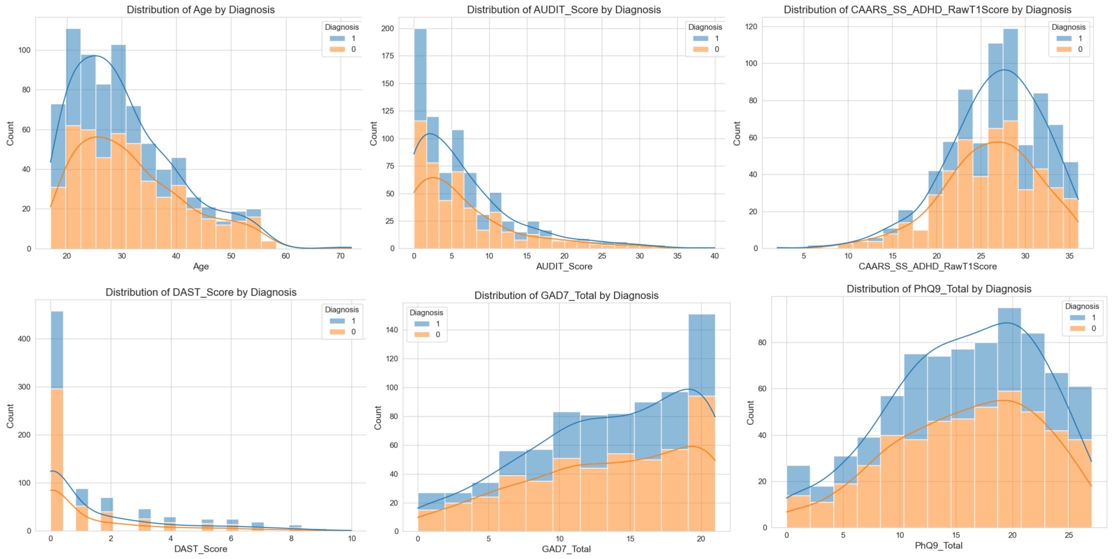

(b)

Figure S2: Comparative distributions of categorical and continuous predictors by diagnostic group. (a) Bar charts for categorical variables: DAST substance-use category, EQ-5D-3L quality-of-life dimensions (Anxiety/Depression, Mobility, Pain/Discomfort, Self-Care, Usual Activities), Gender, HELPS brain-injury screen outcome, and MDQ bipolar screen outcome; (b) Overlaid histograms and density plots for continuous variables: age, AUDIT alcohol-use score, CAARS SS ADHD raw total score, DAST score, GAD-7 anxiety score, and PHQ-9 depression score, stratified by ADHD (orange) and non-ADHD (blue) diagnoses.

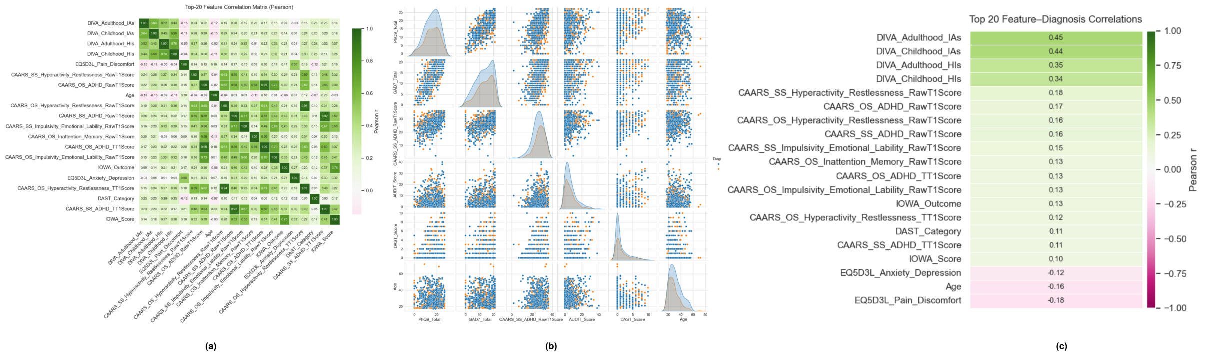

Figure S3: Correlation analyses among top features: (a) Pearson correlation heatmap for the 20 most influential predictors; (b) pairplot of selected continuous variables with histograms and scatterplots colored by diagnosis; (c) bar chart of Pearson correlation coefficients between each top feature and ADHD diagnosis, ordered by absolute value.

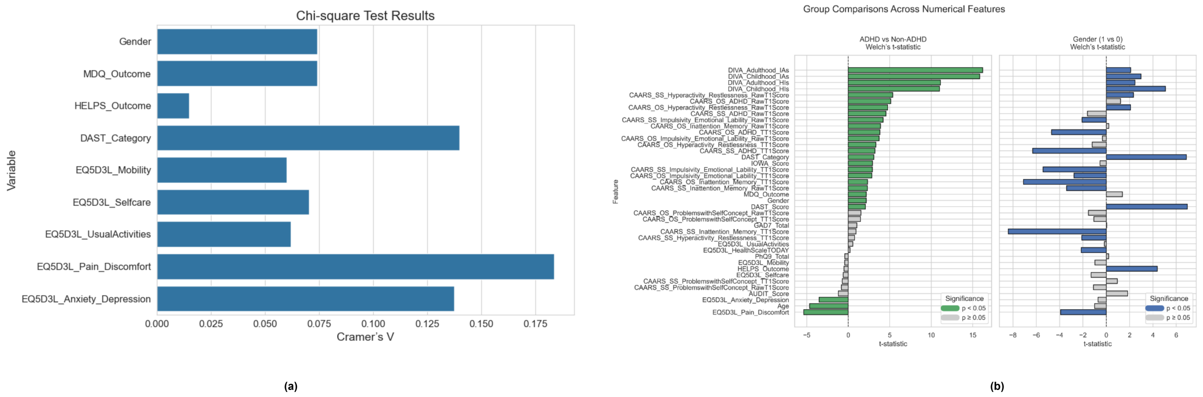

Figure S4: Statistical comparisons of diagnostic groups: (a) Cramér's V from chi-square tests for categorical features versus ADHD diagnosis; (b) Welch's t-statistics comparing numerical features for ADHD vs. non-ADHD (left) and for gender differences (right), with significant ($p < 0.05$) values shaded.

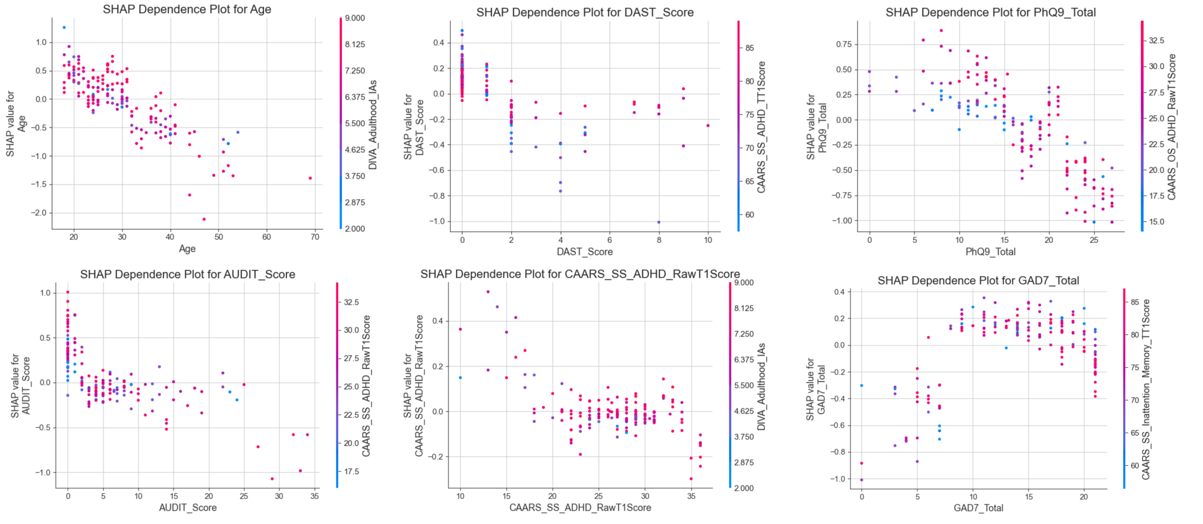

Figure S5: SHAP dependence plots for non-ADHD (class 0): (a) Age; (b) DAST Score; (c) PHQ-9 Total; (d) AUDIT Score; (e) CAARS SS ADHD RawT1 Score; (f) GAD-7 Total.

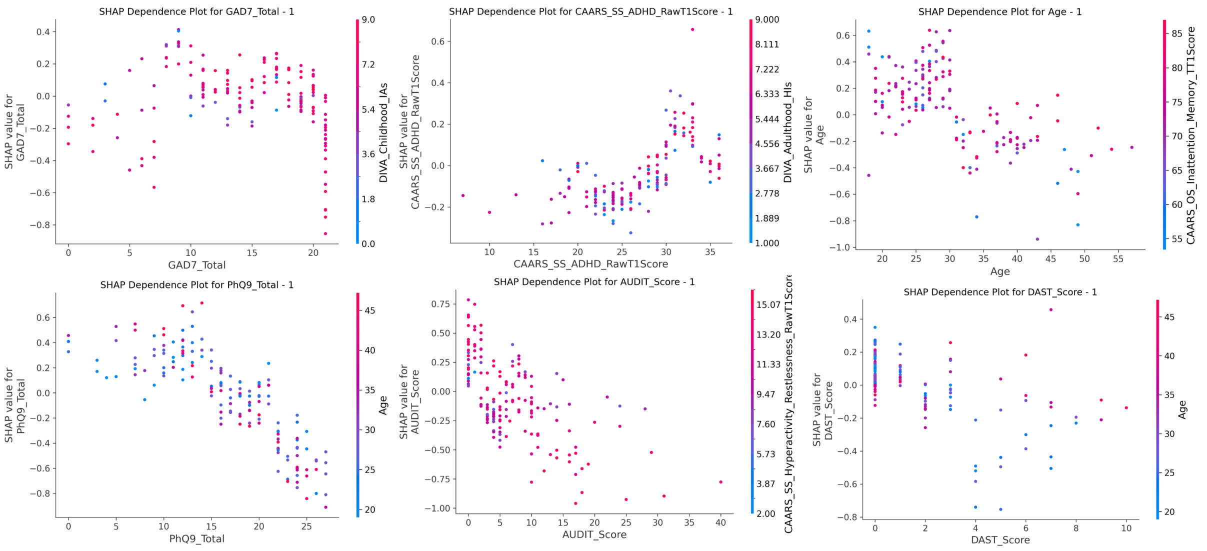

Figure S6: SHAP dependence plots for ADHD (class 1): (a) GAD-7 Total; (b) AUDIT Score; (c) CAARS SS ADHD RawT1 Score; (d) PHQ-9 Total; (e) Age.

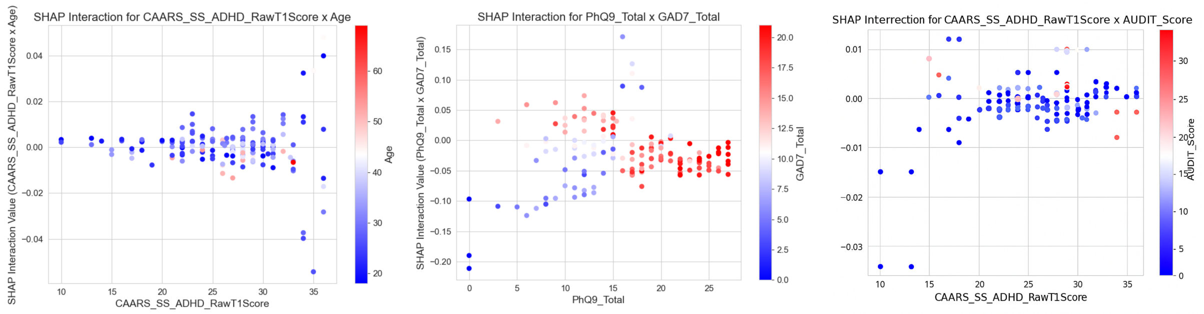

Figure S7: SHAP interaction plots for non-ADHD (class 0): (a) CAARS × Age; (b) PHQ-9 × GAD-7; (c) CAARS × AUDIT.

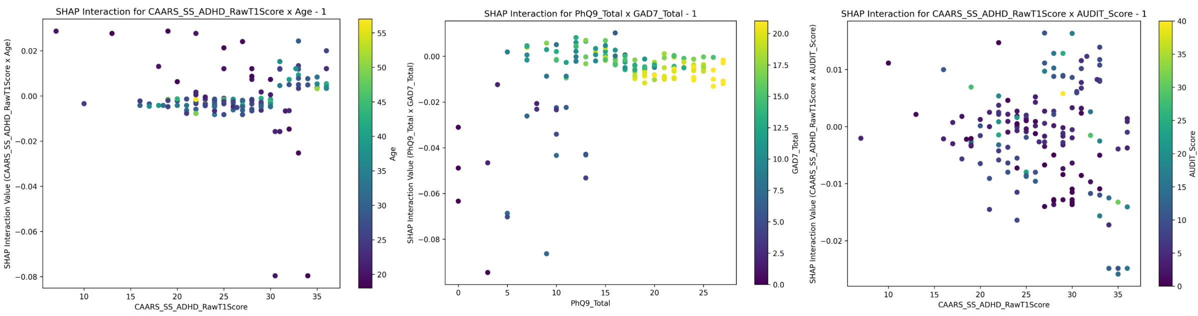

Figure S8: SHAP interaction plots for ADHD (class 1): (a) CAARS × Age; (b) PHQ-9 × GAD-7; (c) CAARS × AUDIT.

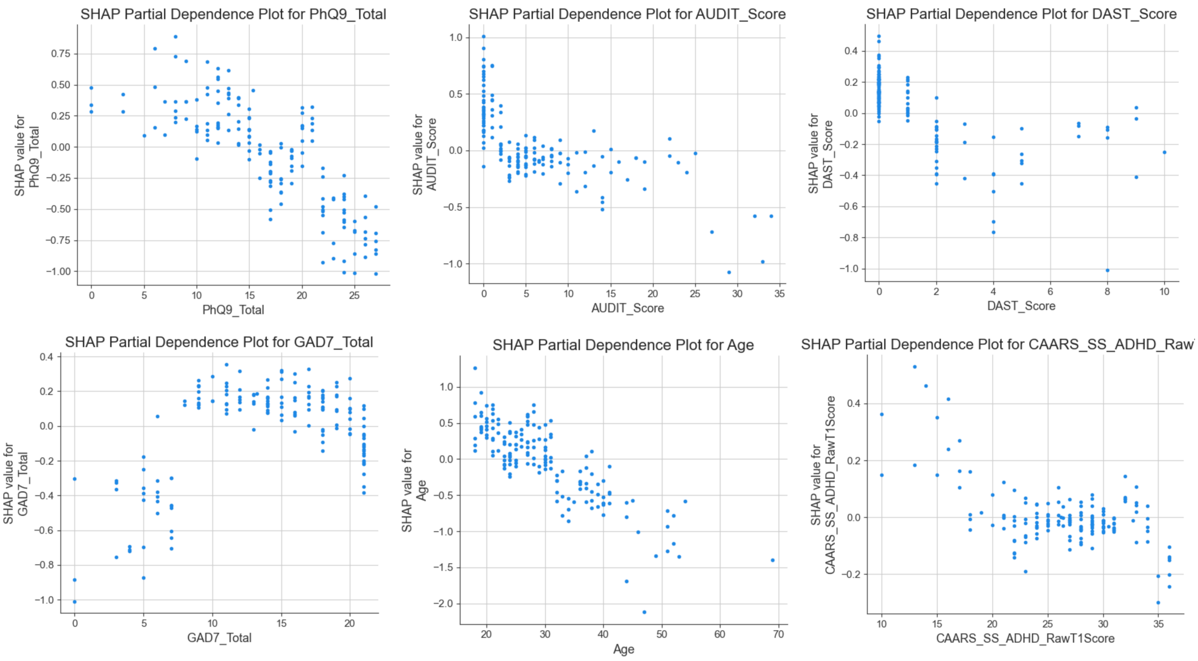

Figure S9: SHAP partial dependence plots for non-ADHD (class 0): (a) PHQ-9 Total; (b) AUDIT Score; (c) DAST Score; (d) GAD-7 Total; (e) Age; (f) CAARS SS ADHD RawT1 Score.

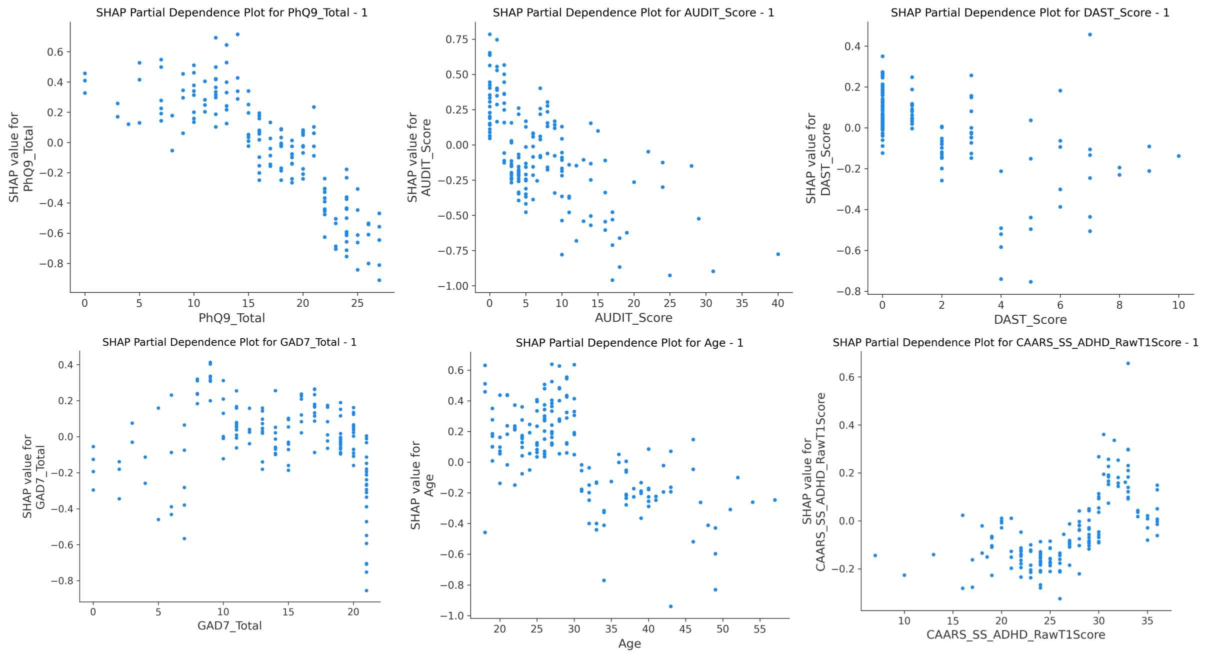

Figure S10: SHAP partial dependence plots for ADHD (class 1): (a) PHQ-9 Total; (b) AUDIT Score; (c) DAST Score; (d) GAD-7 Total; (e) Age; (f) CAARS SS ADHD RawT1 Score.
